# Generation of primordial germ cell-like cells by two germ plasm components, *dnd1* and *nanos3*, in medaka (*Oryzias latipes*)

**DOI:** 10.1016/j.isci.2025.111977

**Published:** 2025-02-08

**Authors:** Toshiya Nishimura, Takafumi Fujimoto

**Affiliations:** 1 Faculty of Fisheries Sciences, Hokkaido University, 3-1-1 Minato, Hakodate, Hokkaido 041-8611, Japan

**Keywords:** Zoology, Genetics, Developmental genetics, Molecular genetics, Developmental biology, Genomics

## Abstract

In teleost fish, primordial germ cells (PGCs), the precursors of eggs and sperm, develop from cells that inherit maternal germ plasm (GP). Although numerous GP component genes have been identified, the minimum set of genes essential for germline formation remains unknown. Herein, we provide evidence that *dnd1* and *nanos3* synergistically induce PGC-like cells from the blastomeres of medaka embryos. One-cell stage embryos injected with *dnd1* and *nanos3* mRNA (DN-OE) showed developmental arrest before gastrulation and upregulation of PGC markers. Transplantation experiments revealed that most transplanted DN-OE blastomeres migrated into the gonadal ridge of host embryos, resulting in the production of functional eggs and sperm. Furthermore, the combination of genome editing and PGC induction techniques successfully generated transgenic knock-in medaka, demonstrating enhanced, precise, and stable gene integration. Identifying essential GP genes for PGC formation advances our understanding of germ cell development mechanisms and their biotechnological applications.

## Highlights

• Germ plasm components, *dnd1* and *nanos3*, are synergistically involved in PGC formation
• Overexpression of *dnd1* and *nanos3* induces PGC-like cells (iPGC) in medaka embryos
• iPGCs develop into functional sperm and eggs
• iPGCs-mediated genome editing enhances the generation of knock-in transgenic fish

## Graphical abstract

**Figure:**
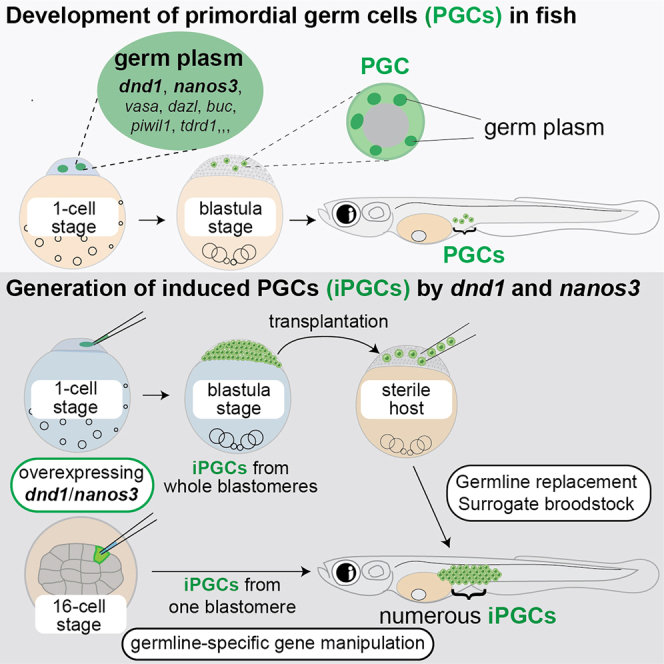

## Introduction

Primordial germ cells (PGCs) are the precursors of eggs and sperm and can transfer genetic materials to the next generation. In animals, PGC formation has two modes: epigenesis and preformation.^1^ In the mode of epigenesis, the germline cell fate is determined by inductive signals from another tissue. In mammals, inductive signals sufficient for PGC formation have been identified,^2^ which underlie the basis for induction techniques of PGCs from embryonic stem cells and induced pluripotent stem cells.^3^^,^^4^ In the mode of preformation, the cells inheriting germ cell fate determinants or germ plasm (GP) accumulated in eggs become PGCs. In teleost fish, the component genes of GP have been identified, such as *vasa*,^5^ *piwi*,^6^^,^^7^ *tdrd*,^8^ *dazl*,^9^^,^^10^ *dead end1* (*dnd1*),^11^^,^^12^ *nanos3*,^13^^,^^14^ and *bucky ball* (*buc*),^15^ but a minimal set of GP components that are sufficient for PGC formation has not been identified.

Several studies have explored inducing PGCs from blastomeres of fish embryos through the overexpression of GP genes. In zebrafish (*Danio rerio*) and medaka (*Oryzias latipes*), embryos with overexpression of *buc* result in an increase in the number of germ cells.^15^^,^^16^ The BUC protein is suggested to function by recruiting other GP components.^15^ Consequently, the overexpression of BUC can induce ectopic formation of GP, resulting in an increase in the number of PGCs. In medaka, the overexpression of *dnd1*, an RNA-binding protein gene essential for germ cell maintenance,^11^^,^^17^ increases the number of germ cells.^12^ However, overexpression of a single GP component appeared to induce PGCs from a subset of blastomeres, suggesting that other components are required for PGC formation. Recently, nine GP components, *vasa*, *dazl*, *piwil1*, *dnd1*, *nanos3*, *tdrd6*, *tdrd7a*, *dazap2*, and *buc*, were shown to efficiently induce PGC-like cells, named iPGCs, from zebrafish blastomeres.^18^ This technique has great potential for applications in genome editing and germline replacement.^18^ However, whether all these factors are required or only a few key factors are sufficient for iPGC formation remains unknown.

The medaka is an experimental model fish suitable for genetic analyses and embryonic manipulation.^19^ The identification of the sex determination gene on the Y chromosome, *DMY*/*dmrt1by*, makes medaka advantageous for studies on reproductive biology, including gonadal sex differentiation and germ cell development.^20^^,^^21^ In this study, we aimed to identify the minimal set of GP components sufficient for PGC formation using medaka. We found that two components, *dnd1* and *nanos3*, synergistically generated iPGCs from blastomeres of medaka embryos.

## Results

### Knockdown of only two factors, *dnd1* and *nanos3*, efficiently induced germ cell-deficient medaka

We previously demonstrated that the CRISPR-Cas13d system was effective for the knockdown of two GP genes, *dnd1* and *nanos3*, to generate germ cell-deficient medaka.^22^ In this system, Cas13d, a CRISPR-Cas RNA endonuclease guided by gRNAs, targets RNAs to be cleaved and degraded.^23^^,^^24^ We initially performed a knockdown screening of GP and germ cell-related genes to identify factors required for germ cell formation using the CRISPR-Cas13d system. We selected 30 representative germline-related genes that are conserved among vertebrates (Table S1)^25^^,^^26^ and designed three gRNAs for each gene using the cas13design tool.^27^^,^^28^ The requirement for germ cell formation and/or maintenance was assessed based on the rate of germ cell-deficient medaka embryos. Notably, the knockdown of only two factors, *dnd1* and *nanos3*, resulted in nearly 100% germ cell depletion in medaka (Table S1).

### *dnd1* and *nanos3* are sufficient to induce PGC-like cells in medaka

**Figure 1:**
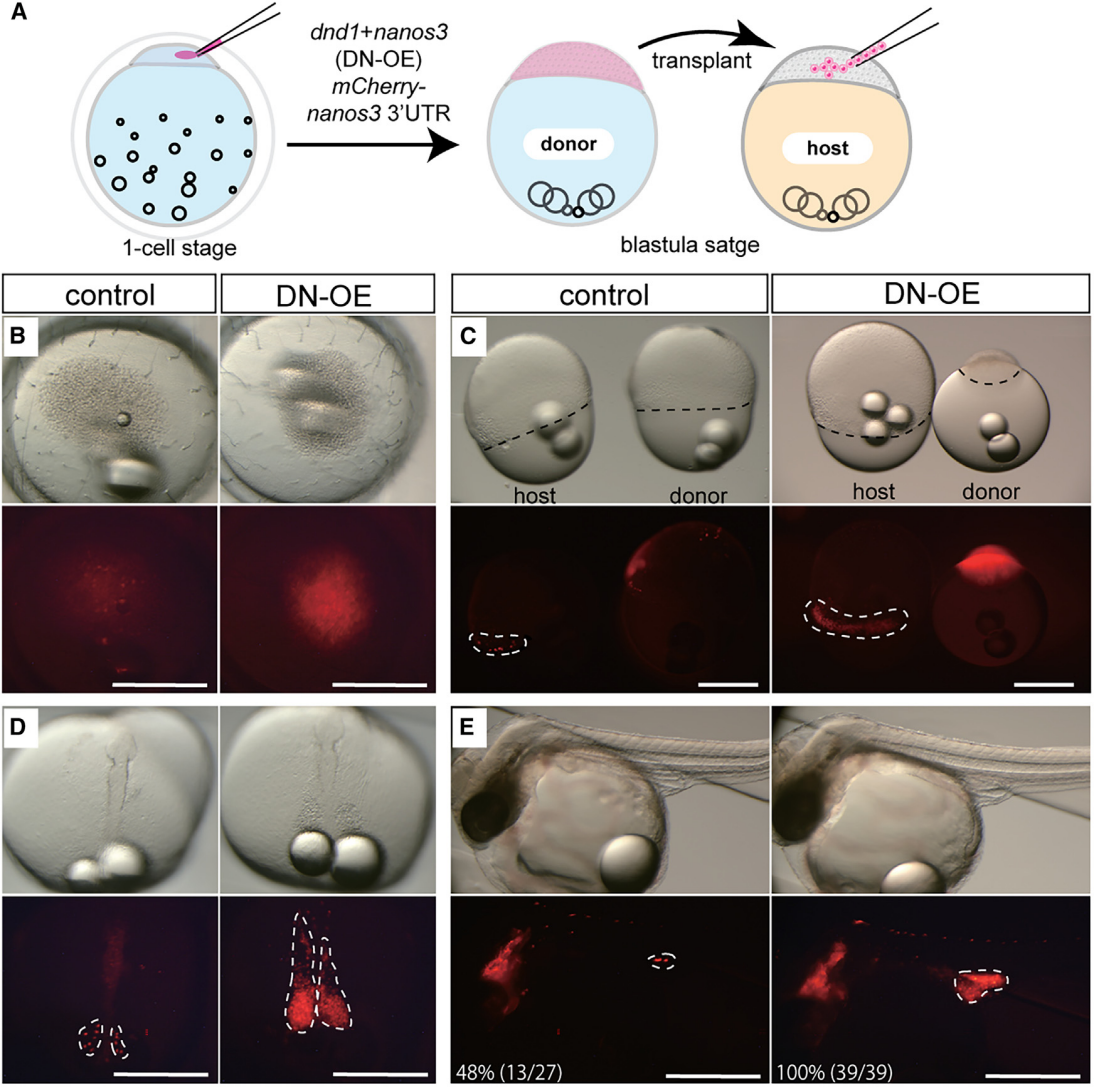
*dnd1* and *nanos3* were sufficient to induce PGC-like cells in medaka (A) Schematic representation of the transplantation experiment using *dnd1* and *nanos3* overexpressed (DN-OE) blastula cells. At the one-cell stage, *dnd1* and *nanos3* mRNAs were injected alongside *mCherry-nanos3* 3′ UTR mRNA for PGC visualization. Blastomeres at the blastula stage were transplanted into wild-type hosts at the same stage. (B) Control and DN-OE donor embryos at the blastula stage. In controls, a subset of PGCs was visualized by mCherry, whereas most blastomeres exhibited high levels of mCherry signals in DN-OE embryos. (C) Host and donor embryos at the gastrula stage (50% epiboly, with margins indicated by black dotted lines). In both control and DN-OE embryos, mCherry-positive PGCs migrated toward the marginal zone of epiboly (encircled by white dotted lines). DN-OE donor embryos were developmentally arrested before the gastrula stage. (D) Chimeric embryos at stage 17 (early neurula stage). mCherry-positive PGCs (white dotted lines) are aligned bilaterally along the embryonic bodies. (E) Germline chimera at stage 35, showing mCherry-positive PGCs reaching the gonads (white dotted lines). The rate of germline chimeras was significantly higher with DN-OE blastomeres (100%) than with controls (48%), as evaluated by Fisher’s exact test (*p* < 0.01). The number in parentheses indicates the number of embryos examined. Scale bars: 500 μm. Refer also to Table S1.

To determine whether *dnd1* and *nanos3* were sufficient for germline formation, we overexpressed *dnd1* and *nanos3* in medaka embryos. The translation of germline transcripts such as *vasa*, *tdrd7*, and *nanos3* is regulated by the 3′ UTR.^29^^,^^30^^,^^31^ In somatic cell lineages, these germline transcripts are degraded by miRNAs.^32^^,^^33^ We replaced *nanos3* and *dnd1* 3′ UTR with *SV40pA* (Figures S1A and S1B) and confirmed that the resultant *nanos3*-*SV40pA*, but not *dnd1*-*SV40pA*, was significantly stabilized in the embryos (Figure S1C). Although we found the higher stability of *dnd1* 3′ UTR compared with *SV40pA*, we used *SV40pA* for subsequent experiments to equalize the stabilization of *dnd1* and *nanos3* mRNA. The *nanos3*-*SV40pA* and *dnd1*-*SV40pA* mRNA were injected into embryos at the one-cell stage (DN-OE: DND1 and NANOS3 OverExpressed) together with *mCherry*-*nanos3* 3′ UTR mRNA for visualization of PGCs (Figure 1A). In wild-type controls at the blastula stage (stage 10),^34^ a subset of PGCs was visualized by mCherry (Figure 1B). In contrast, DN-OE embryos at the blastula stage showed an upregulation of the mCherry signal in whole blastomeres (Figure 1B) and were developmentally arrested before gastrulation.

To further characterize the fate of DN-OE blastomeres, we transplanted a clutch of blastomeres containing >50 cells at the blastula stage into a wild-type host at the same stage (Figure 1A). In the control blastula transplantation experiment, a subset of transplanted cells fated to become PGCs migrated toward the marginal zone during epiboly (Figure 1C) and were bilaterally aligned along the embryonic body by the early neurula stage (stage 17, Figure 1D).^35^ Surprisingly, the majority of transplanted DN-OE blastomeres migrated in the same manner and eventually arrived at the gonads (Figures 1C–1E). The rate of DN-OE blastomeres arriving at gonads (germline chimera) was 100% (*n* = 39), which was significantly higher than that of control PGCs (Figure 1E, 48%, *n* = 27, *p* < 0.01). These results indicated that DN-OE blastomeres become PGC-like cells that can migrate to the genital ridge. These PGC-like cells were named induced-PGCs (iPGCs), according to a previous study.^18^

### iPGCs generated by *dnd1* and *nanos3* differentiate into functional eggs and sperm

**Figure 2:**
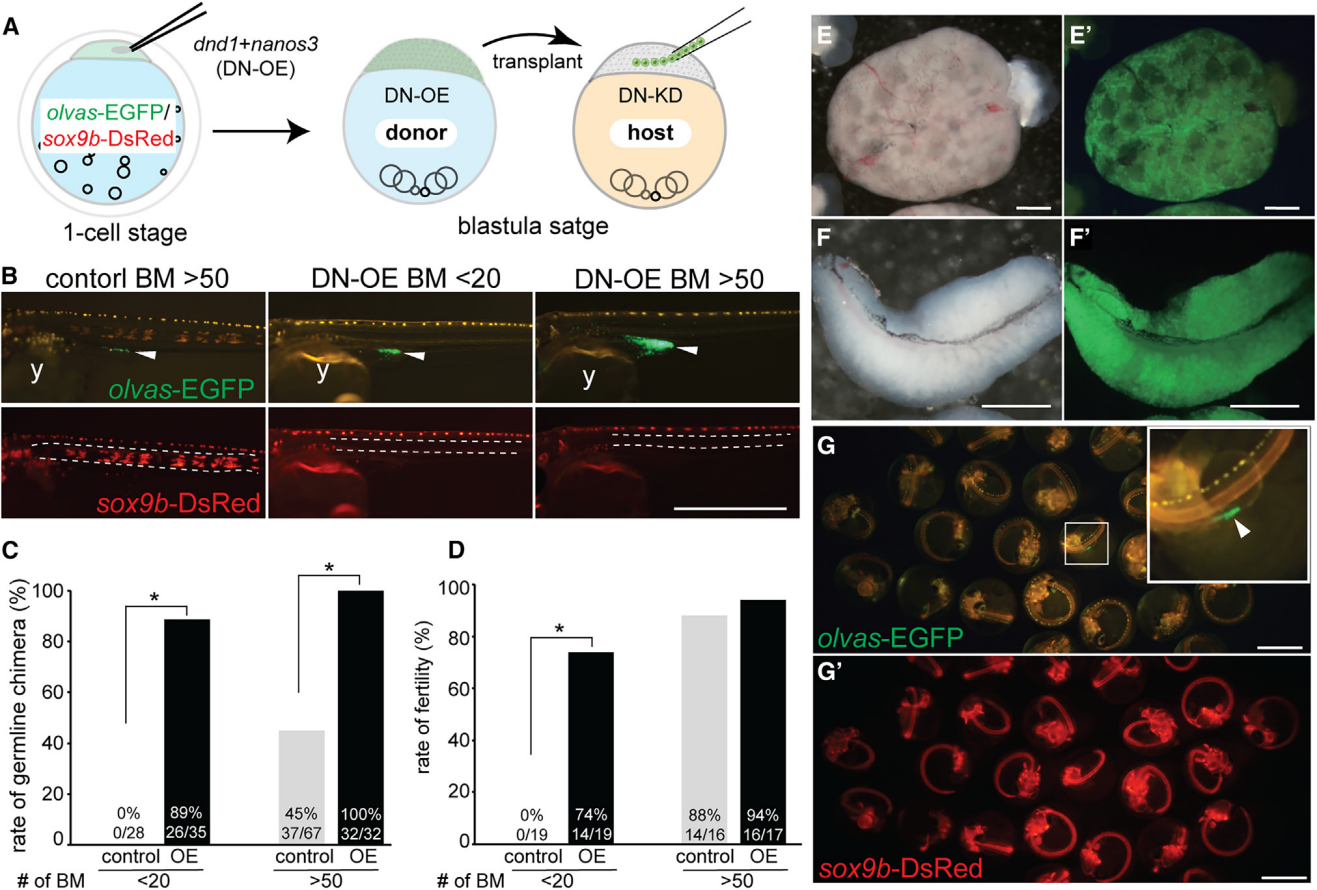
iPGCs generated by *dnd1* and *nanos3* differentiated into functional eggs and sperm (A) Schematic representation of the transplantation experiment using *dnd1* and *nanos3* overexpressed (DN-OE) blastomeres. *dnd1* and *nanos3* mRNAs were injected into *sox9b*-DsRed/*olvas*-EGFP transgenic embryos at the one-cell stage. DN-OE blastomeres at the blastula stage were transplanted into *dnd1* and *nanos3* knockdown (DN-KD) embryos at the same stage. (B) Germline chimera at 7 days post-fertilization (dpf; just before hatching stage). A total of <20 or >50 blastomeres (BM) were transplanted. Arrowheads indicate the gonadal region. The space between white dotted lines indicates the notochord (y: yolk). (C) Rate of germline chimeras using control and DN-OE (OE) blastomeres (BM). (D) Fertility rates of 3-month-old adult chimeras. Germline chimeras confirmed at 7 dpf were used for fertility evaluation. Non-germline (*olvas*-EGFP negative) chimeras generated by transplanting <20 control BM were also assessed for fertility evaluation. Asterisks indicate significant differences, as evaluated by Fisher’s exact test (*p* < 0.01). (E–F) The ovary (E) and testis (F) from germline chimeras derived from DN-OE blastomeres. *olvas-*EGFP signals were detected in both gonads (E′ and F′). (G) Progeny from DN-OE germline chimeras. All embryos showed *olvas*-EGFP (G, arrowhead in the inset) and *sox9b*-DsRed signals (G′). Scale bars: 1 mm. Refer also to Table S2.

To confirm whether iPGCs generated by DN-OE could produce functional eggs and sperm, we used *sox9b*-DsRed/*olvas*-EGFP transgenic medaka^36^ as DN-OE donor embryos. In this transgenic line, gonadal somatic cells and the notochord were labeled with DsRed, and germ cells were labeled with EGFP.^36^ To determine whether transplanting fewer cells could efficiently generate germline chimeras, we transplanted <20 or >50 DN-OE blastomeres into germ cell-deficient hosts by *dnd1* and *nanos3* knockdown (DN-KD) using the CRISPR-Cas13d system (Figure 2A). When <20 blastomeres were transplanted into host embryos, no germline chimeras were obtained from control donor cells, whereas 89% of embryos developed into germline chimeras from DN-OE donor cells (Figures 2B and 2C). When >50 blastomeres were transplanted, 45% and 100% of germline chimeras were obtained from control and DN-OE donor cells, respectively (Figures 2B and 2C). The rate of germline chimeras was significantly higher when using DN-OE blastomeres than control blastomeres (Figure 2C, *p* < 0.01). In the control germline chimeras generated by transplanting >50 blastomeres, *sox9b*-DsRed signals were observed at the notochord, in addition to *olvas*-EGFP signals in the gonads at the hatching stage, suggesting transplanted cells differentiated into both somatic and germline lineages (Figure 2B). In contrast, germline chimeras generated from DN-OE blastomeres showed no *sox9b-*DsRed signals in the notochord (Figure 2B), suggesting that most DN-OE blastomeres contributed to iPGCs.

Germline chimeras generated from DN-OE blastomeres developed into both females and males with *olvas*-EGFP-positive ovaries and testes, respectively (Figures 2E and 2F, Table S2), and 74% (<20 blastomeres) and 94% (>50 blastomeres) of DN-OE germline chimera were fertile, which was comparable to the control germline chimera (88%, >50 blastomeres, Figure 2D). All F1 progeny from the female and male DN-OE germline chimeras crossed with wild-type fish showed *olvas*-EGFP and *sox9b*-DsRed signals (Figure 2G), indicating that iPGCs generated from DN-OE blastomeres developed into functional eggs and sperm.

**Figure 3:**
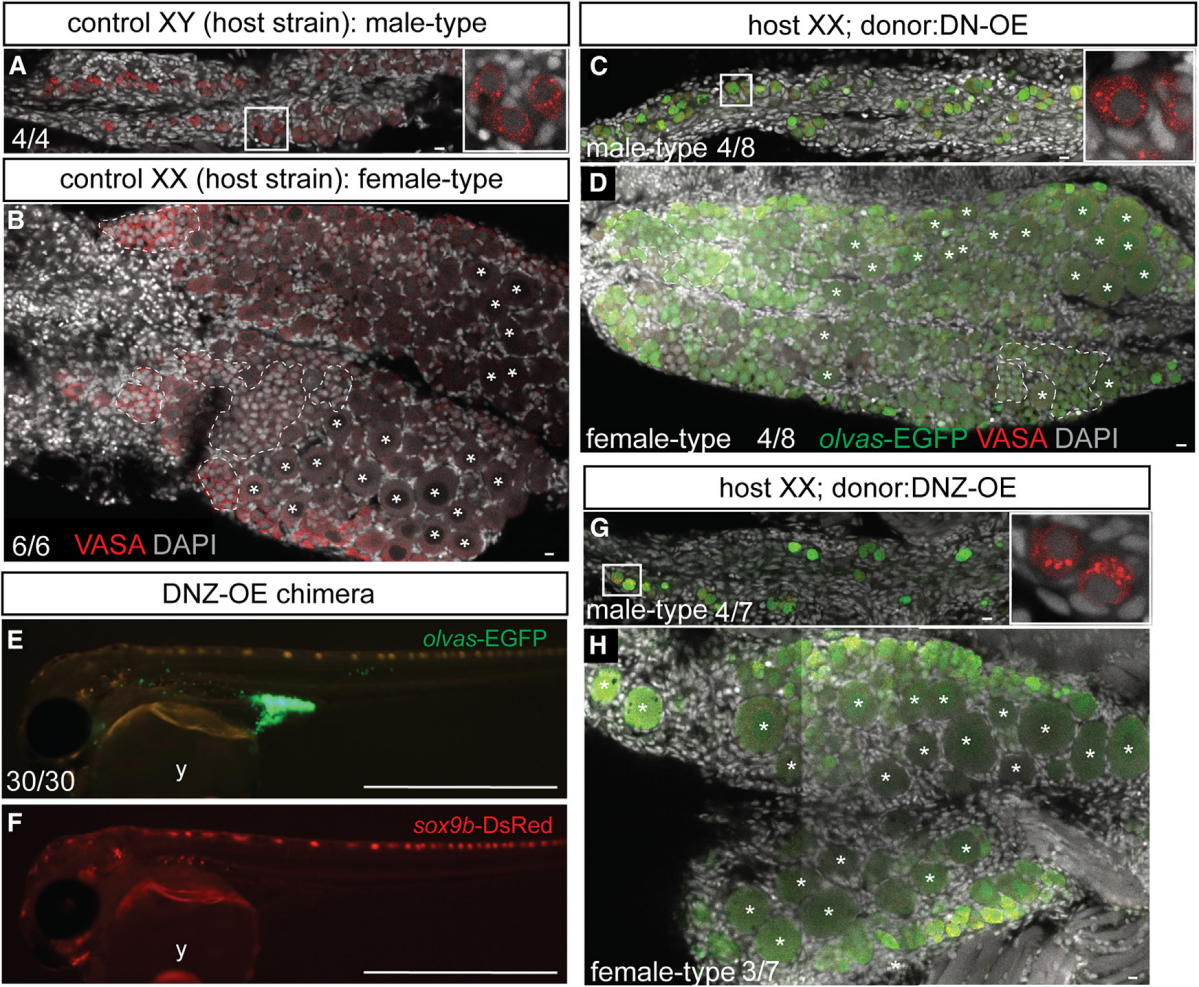
Observation of male- and female-type gonads in DN-OE chimeric XX medaka at 10 days post-hatching (A) Control XY male gonad at 10 days post-hatching (dph) from host Cab strain medaka. Only single isolated germ cells (type I, red) were observed. The area within the white square is magnified in the inset. (B) Control XX female gonad at 10 dph. Numerous cystic germ cells (dotted lines) and oocytes (asterisks) were observed. (C) Male-type gonad in *dnd1* and *nanos3* overexpressed (DN-OE) XX chimera. Only type I germ cells were observed, with *olvas*-EGFP-positive cells (green) indicating iPGCs derived from DN-OE donors. Type I germ cells in the white square are magnified in the inset, expressing endogenous VASA protein (red). (D) Female-type gonad from DN-OE XX chimera. The gonad was filled with numerous EGFP-positive germ cells, including cystic germ cells (dotted lines) and oocytes (asterisks). (E and F) Germline chimera generated by transplanting *dnd1*, *nanos3,* and *dazl* overexpressed (DNZ-OE) donor blastomeres. All chimeras showed abundant donor-derived *olvas*-EGFP-positive iPGCs (E) and a minimal contribution of *sox9b*-DsRed-positive somatic cells (F) (y: yolk). (G) Male-type gonad from DNZ-OE XX chimera. Donor-derived *olvas-*EGFP-positive type I germ cells (green) are shown, with the white square magnified in the inset, expressing endogenous VASA protein (red). (H) Female-type gonad from DNZ-OE XX chimera. Donor-derived *olvas*-EGFP-positive iPGCs differentiated into oocytes (asterisks). Nuclei were stained with DAPI (gray). Scale bars: 10 μm in A–D, G, and H; and 1 mm in E and F. Refer also to Table S2.

We noticed that 45% of XX DN-OE chimeras (>50 blastomeres) showed sex reversal in males (Table S2). Considering that the DN-OE chimera showed a large number of germ cells in the gonads at the hatching stage (Figure 2B), this result was unexpected because medaka showed sex reversal depending on the number of germ cells; few germ cells and a large number of germ cells induced sex reversal from female-to-male and male-to-female, respectively.^37^^,^^38^^,^^39^^,^^40^ To obtain insights into the causes of sex reversal, we observed DN-OE chimeric gonads at 10 days post-hatching (dph), when sexual differences between XX and XY gonads were obvious in wild-type.^41^ Wild-type XY gonads at 10 dph had only single isolated germ cells surrounded by gonadal somatic cells (Figure 3A) that are known as type I germ cells or germline stem cells.^42^ In contrast, control XX gonads initiated oogenesis and contained many cystic (type II) germ cells and oocytes (Figure 3B). Notably, DN-OE chimeric XX gonads at 10 dph exhibited both male-type gonads with only type I germ cells (Figure 3C) and female-type gonads with numerous oocytes (Figure 3D). In the male-type gonads, the number of DN-OE germ cells drastically decreased, and the initiation of gametogenesis was suppressed. A few chimeras eventually lost whole germ cells during gonadal development as germ cell-deficient gonads were observed in the adult stage (Table S2). *dazl* is a critical gene for the initiation of gametogenesis in both mammals^43^ and fish.^44^ Transplantation of >50 donor blastomeres with overexpression of *dazl*, along with *dnd1* and *nanos3* (DNZ-OE), resulted in 100% generation of germline chimeras with a large number of *olvas-*EGFP-positive iPGCs (Figures 3E and 3F). However, the addition of *dazl* did not recover the initiation of gametogenesis, as approximately 50% of DNZ-OE chimeric gonads showed male-type gonads with only type I germ cells (Figures 3G and 3H). Consistent with this observation, female-to-male sex reversal was also observed in the DNZ-OE chimera; however, most adult chimeric fish were fertile (Table S2), suggesting that a few iPGCs at 10 dph eventually recovered gametogenesis by the sexually mature stage. Therefore, although iPGCs generated by DN-OE and DNZ-OE developed into functional eggs and sperm, iPGCs likely differ in their characteristics from wild-type PGCs in terms of their potential to initiate gametogenesis and feminization of gonads.

### *dnd1* and *nanos3* synergistically generated iPGCs

**Figure 4:**
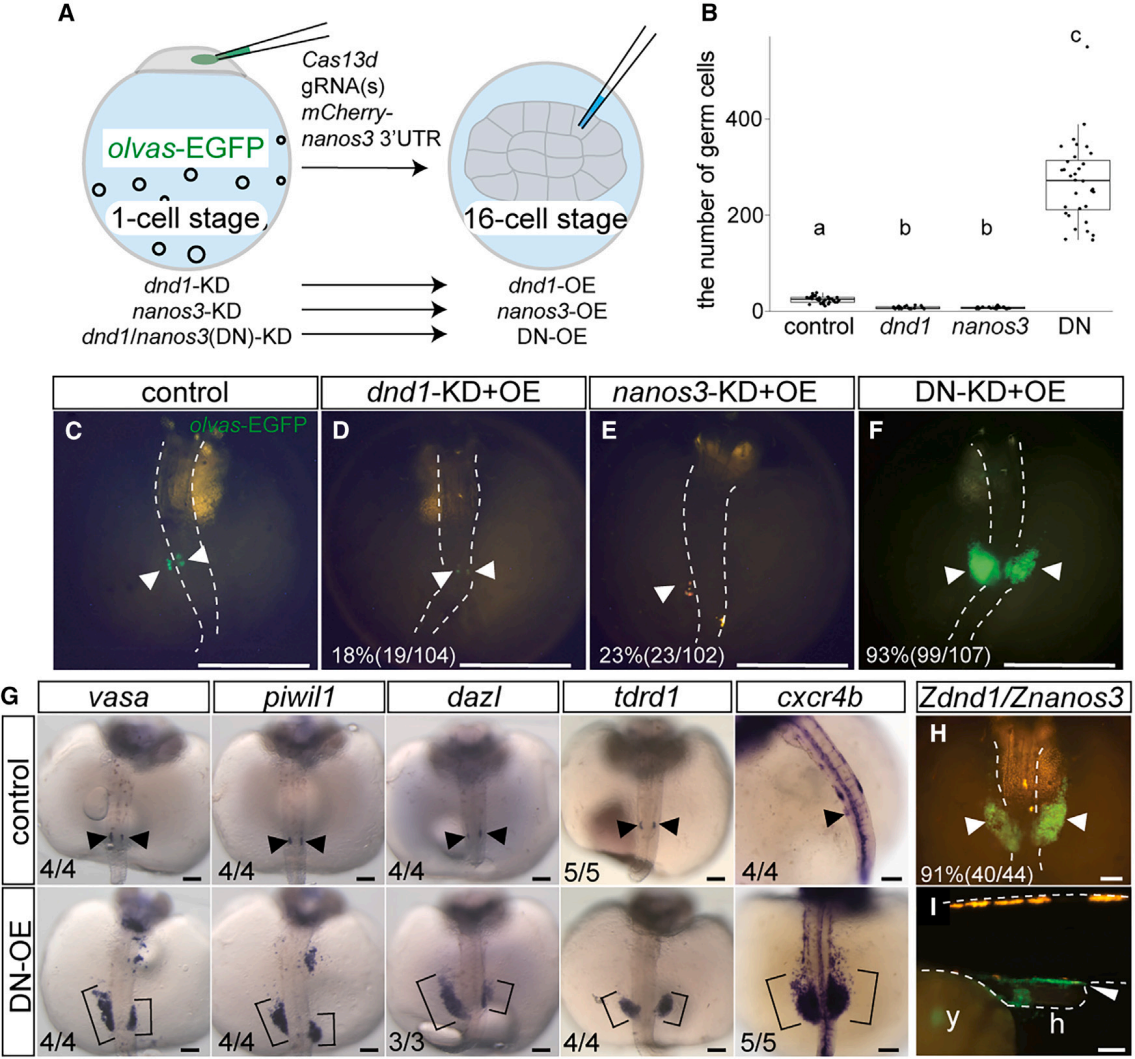
Induction of iPGCs from a specific blastomere (A) Schematic representation of iPGC generation from a specific blastomere. At the one-cell stage of *olvas*-EGFP transgenic medaka, the knockdown (KD) of *dnd1*, *nanos3*, or a combination of *dnd1* and *nanos3* (DN) was performed using the CRISPR-Cas13d system. At the 16-cell stage, *dnd1*, *nanos3*, or *dnd1*/*nanos3* mRNA was injected into the corner of a blastomere for overexpression (OE). (B)The number of germ cells in control (*n* = 29), *dnd1*-OE (*n* = 19), *nanos3*-OE (*n* = 23), and DN-OE embryos (*n* = 31) at stage 29. Each dot indicates one individual. Only embryos with germ cells were evaluated for quantification. Note that the number of DN-OE iPGCs was estimated based on the area of EGFP-positive iPGCs (see STAR Methods). Different letters represent statistical significance (*p* < 0.01) between groups, which were analyzed by the Kruskal–Wallis test followed by Dunn’s test. (C) In control embryos at stage 29, a pair of clusters of *olvas*-EGFP-positive germ cells (arrowheads) was localized bilaterally to the hindgut. (D and E) Injection of *dnd1* or *nanos3* mRNA alone into *dnd1* or *nanos3*-KD embryos resulted in minimal recovery of *olvas*-EGFP germ cells. The “%” indicates the rate of embryos with at least one EGFP-positive germ cell. (F) Injection of both *dnd1* and *nanos3* mRNAs (DN-OE) resulted in the generation of large germ cell clusters (arrowheads). The “%” indicates the rate of embryos with large germ cell clusters. (G) Expression analyses of germ plasm genes by *in situ* hybridization. *vasa*, *piwil1*, *dazl*, *tdrd1*, and *cxcr4b* were expressed in iPGCs generated from a DN-OE blastomere. Arrowhead and square brackets indicate control PGCs and DN-OE iPGCs, respectively. (H and I) Generation of iPGCs by *dnd1* and *nanos3* derived from zebrafish. Medaka embryos at stage 29 (H) and hatching stage (I). Arrowheads indicate *olvas*-EGFP-positive iPGC (y: yolk, h: hindgut). Embryonic bodies are outlined by white dotted lines (C–F and H–I). Scale bars: 100 μm. Refer also to Figure S2 and Table S3.

To determine whether both *dnd1* and *nanos3* are essential for generating iPGCs, we assessed the iPGC forming capacity by injecting *dnd1* or *nanos3* mRNA alone or together into one blastomere of 16-cell stage embryos where endogenous *dnd1* and/or *nanos3* had been depleted by knockdown using the CRISPR-Cas13d system (Figure 4A).^22^ The gRNA target sites of the injected *dnd1* and *nanos3* mRNA were mutated to protect against degradation (Figures S1A and S1B). We used the progeny from wild-type females crossed with *olvas*-EGFP males to monitor the zygotic expression of *vasa*. When *dnd1* or *nanos3* alone was overexpressed in one blastomere of 16-cell stage embryos, in which endogenous *dnd1* or *nanos3* had been knocked down at the one-cell stage, only 18% and 23% of embryos recovered a few germ cells, respectively (Figures 4D and 4E). The average number of PGCs was 3.6 and 3.3 in *dnd1* and *nanos3*-OE embryos, respectively, which was significantly lower than the average of 20.4 PGCs in the control embryos at stage 29 (Figures 4B–4E). In contrast, co-injection of *dnd1* and *nanos3* mRNAs led to 93% of embryos generating a significantly large number of iPGCs, with an average of 275, compared with control, *dnd1*-OE*,* and *nanos3*-OE embryos. (Figures 4B and 4F). The iPGCs strongly expressed zygotic *olvas*-EGFP (Figure 4F). Further expression analysis through *in situ* hybridization revealed that GP genes, *vasa*,^29^ *piwil1*,^7^ *dazl*,^9^ and *tdrd1*^8^ were expressed in iPGCs (Figure 4G). In addition, the chemokine receptor *cxcr4b* was expressed in iPGCs (Figure 4G), suggesting proper migration depending on its ligand SDF1.^35^ Interestingly, we also found that *nanos3* and *dnd1* derived from zebrafish generated iPGC in medaka (Figure 4H), which eventually arrived at the gonads (Figure 4I). Although approximately half of the XX fish with iPGCs showed female-to-male sex reversal in the adults, more than 90% of the fish became fertile (Table S3), suggesting that iPGCs generated by zebrafish *dnd1* and *nanos3* developed into functional sperm and eggs in medaka. These data suggest that *dnd1* and *nanos3* synergistically generate iPGCs, which may be conserved in a wide variety of teleost fish.

Nine GP genes (*vasa*, *dazl*, *piwil1*, *dnd1*, *nanos3*, *tdrd6*, *tdrd7a*, *dazap2*, and *buc*) were used to generate iPGCs in zebrafish.^18^ To confirm the essential roles of *dnd1* and *nanos3* among the nine GPs for the generation of iPGCs in medaka, we evaluated the iPGC-forming capacity by injecting mRNAs of the seven GP genes (*vasa*, *dazl*, *piwil1*, *tdrd6*, *tdrd7a*, *dazap2*, and *buc*) along with either *dnd1* or *nanos3* into one blastomere located at the corner of the 16-cell stage embryos (Figure S2A). The results clearly showed that the iPGC-forming capacity was drastically reduced when either *dnd1* or *nanos3* was removed from the nine GP genes (Figures S2B–S2D), further supporting the notion that these two components are critical for PGC formation.

### iPGCs are available for genome editing

**Figure 5:**
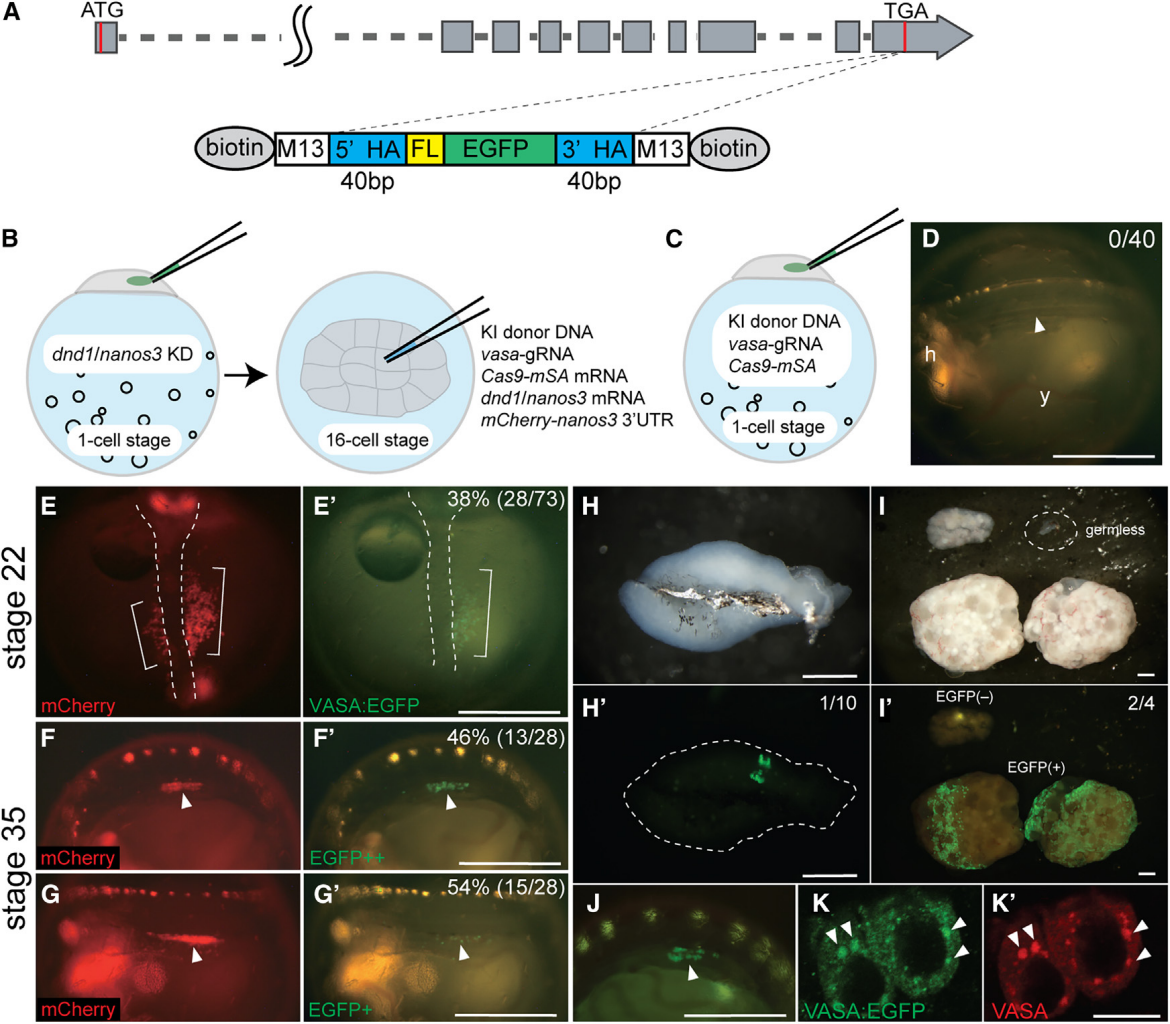
Generation of *vasa:EGFP* knock-in transgenic medaka from iPGCs (A) Schematic representation of the *vasa* gene with the integration site for donor DNA containing 5′ biotinylated 40 bp short homology arms and M13 primer sequences. The 5′ biotin was attached by PCR using 5′ biotinylated M13 forward and reverse primers (see Figure S3). (B) Schematic representation of the injection for knock-in (KI) at the 16-cell stage. At the one-cell stage, endogenous *dnd1* and *nanos3* were depleted by injecting *Cas13d* mRNA and *dnd1* and *nanos3* gRNAs. At the 16-cell stage, KI injectates containing 5′ biotinylated donor DNA, *vasa*-gRNA, *Cas9-mSA* mRNA, and *mCherry-nanos3* 3′ UTR (for visualization of iPGCs) were injected along with *dnd1* and *nanos3* mRNA. (C) A conventional method involving the introduction of KI injectates into one-cell stage embryos. (D) No EGFP-positive embryos were obtained using the conventional method in C. The arrowhead indicates the position of the gonad at stage 35. (E) The KI DN-OE embryos at the somite stage (stage 22). mCherry-positive and EGFP-positive iPGCs are indicated by white brackets. White dotted lines indicate the embryonic body. The “%” indicates the rate of embryos with EGFP-positive iPGCs. (F and G) DN-OE embryos at stage 35, when gonad formation is complete (arrowheads). Among the EGFP-positive embryos, 46% showed a majority of EGFP-positive cells overlapping with mCherry-positive iPGCs (F and F′). Conversely, 54% exhibited EGFP-positive cells sparsely distributed among mCherry-positive iPGCs (G and G′). (H) The testis from F0 male founders (*n* = 10), showing one testis with EGFP-positive cells (H′). (I) Ovaries from F0 female founders (*n* = 4), with two ovaries containing EGFP-positive cells and one gonad with no observed germ cells. (J) F1 embryos at stage 35 from the female founder, with the arrow indicating the position of the gonad with EGFP-positive germ cells. (K) Immunohistochemical staining of VASA:EGFP (K, green) and endogenous VASA (K′, red). Arrows indicate the co-localization of VASA:EGFP and VASA in germ granules. Scale bars: 500 μm in D, E′–G′, and J; 1 mm in H and I; and 10 μm in K. Refer also to Figure S3–S5.

Precise gene integration by homology-directed repair (HDR) using the CRISPR-Cas9 system is a useful technique for endogenous protein tagging and has been developed in vertebrates.^45^^,^^46^ However, to date, endogenous GP protein tagging with a fluorescent protein has not been reported in teleosts. We attempted to generate knock-in (KI) medaka with EGFP fused to the C-terminus of the endogenous VASA protein (VASA:EGFP, Figures S3A–S3C) by injecting KI solutions containing *Cas9-mSA* (monomeric Streptavidin) mRNA,^45^ *vasa-*gRNA, and donor DNA with 5′ biotinylated short homology (40 bp) arm^46^ into one-cell stage embryos, but no embryos with EGFP-positive germ cells were obtained (Figures 5A, 5C and 5D). Subsequently, we investigated the potential for the combination of iPGC induction and genome editing techniques to facilitate the generation of *vasa:EGFP* KI medaka.

First, endogenous *dnd1* and *nanos3* were depleted by knockdown, followed by injection of *Cas9-mSA* mRNA, *vasa*-gRNA, and donor DNA with 5′ biotinylated short homology (40 bp) arm together with *dnd1* and *nanos3* mRNA into one blastomere of 16-cell stage embryos (Figure 5B). Initially, we confirmed that injection into the center of the blastomere at the 16-cell stage resulted in higher efficiency for *EGFP* integration than injection into the corner of the blastomere (Figures S4A–S4B). To prepare KI founders, we injected KI solutions into the center of a blastomere, which resulted in the generation of 73 embryos with iPGCs and the identification of 28 EGFP-positive embryos at stage 22 (Figure 5E). When iPGCs reached the gonads at stage 35, two types of gonads were observed. In one type of gonads, the EGFP-positive cells fully overlapped with the mCherry-labeled iPGC (Figure 5F). The other gonads showed that EGFP-positive cells were sparsely distributed in the iPGC population (Figure 5G). The rate of gonads with full overlap was approximately 50%, which was higher when injections were made into the center of the blastomere compared to injections into the corner (20%) (Figures 5F, S4C, and S4D). Fourteen adult fish survived from EGFP-positive embryos (*n* = 28). Among them, 1/10 testes and 2/4 ovaries showed EGFP fluorescence (Figures 5H and 5I). We successfully obtained F1 EGFP-positive progeny (Figure 5J) from the two females at the rates of 98% (*n* = 85) and 18% (*n* = 45), respectively, which showed precise EGFP integration at the expected *vasa* locus position (Figure S3C). Furthermore, EGFP signals were co-localized with the endogenous VASA protein (Figure 5K). The transgene was stably transmitted across generations, with EGFP signals observed in F4 embryonic gonads and appropriately localized within germ cells (Figure S5). Therefore, the combination of iPGC induction and CRISPR-Cas9 enables the generation of precise and stable protein-tagged transgenic fish.

## Discussion

In this study, we demonstrated that the minimum components of the GP genes, *dnd1* and *nanos3*, synergistically generated iPGCs in medaka. Chimeric analyses have revealed that iPGCs can differentiate into functional eggs and sperm. In addition, by using iPGC induction, we demonstrated the enhancement of precise endogenous protein tagging by CRISPR-Cas9 mediated KI. These findings provide insights into germ cell biology in vertebrates and hold a significant potential for applications in the development of genetic tools using genome editing.

### Comparison of iPGC characteristics with PGC

The iPGCs generated by two GPs, *dnd1* and *nanos3*, in medaka are likely equivalent to those generated by nine GPs in zebrafish^18^ in terms of the capacity for gametogenesis initiation and feminization of gonads. In zebrafish, iPGCs generated by nine GPs decreased in number after gonad formation, leading to an all-male phenotype. In zebrafish, oocytes are critical for the feminization of gonads.^47^ Therefore, the all-male phenotype suggested that iPGCs failed to initiate gametogenesis and produce sufficient oocytes for feminization. Here, we also found that approximately 50% of XX gonads with iPGCs showed a decrease in the number and did not initiate gametogenesis by 10 dph (Figure 3). A few of them eventually lost whole germ cells, leading to infertility in the adult stage (Tables S2 and S3). This reduction likely leads to the female-to-male sex reversal in XX medaka. The failure of the gametogenesis initiation was not recovered by the addition of *dazl*, an essential factor for the initiation of gametogenesis.^43^^,^^44^ Additionally, iPGCs expressed *dazl* during the migration toward the gonads (Figure 4F). These observations suggest the presence of a distinct pathway affecting the initiation of gametogenesis in germ cells.

In the chimeric analyses, we did not examine the genotype (XX or XY) of donor embryos and could not determine whether the sex reversal was due to the XY genotype of iPGCs. Nevertheless, we found that XX embryos with XX iPGCs, which were generated by overexpressing *dnd1* and *nanos3* in one blastomere of the 16-cell stage, also showed approximately 50% female-to-male sex reversal in the adult stage (Table S3). Therefore, the sex reversal of XX fish with iPGCs may not be explained by the presence of the Y chromosome in the iPGCs.

Approximately 50% of XX fish with iPGCs could develop into females in medaka (Tables S2 and S3), which is in contrast to the all-male phenotype of zebrafish.^18^ This may be due to the differences in feminization tendencies by germ cells between zebrafish and medaka. In medaka, PGCs have an inherent feminizing effect on gonads,^44^ and unlike zebrafish, oocytes are not necessary for feminization. It may also be possible that the overexpression of the minimum GP components did not interfere with PGC function to the same extent as the nine GPs used in zebrafish. In summary, iPGCs in both zebrafish and medaka likely differ in their characteristics from wild-type PGCs in terms of their potential for initiation of gametogenesis and feminization of gonads. Further studies, such as gene expression and proteomics analyses, will help understand the differences between iPGCs and normal PGCs in teleost fish.

### Application of iPGCs in germline replacement techniques

Germline replacement by cell transplantation is a useful technique for the generation of maternal-zygotic mutants of embryonic lethal genes^48^^,^^49^ as well as conservation and production of valuable and endangered species, which is known as the surrogate broodstock technique.^50^^,^^51^ Sterilization of the host and isolation of donor germ cells from embryos or gonads are essential for efficiently generating donor gametes. In previous and present studies, we showed that knockdown of either *dnd1* or *nanos3* using the CRISPR-Cas13d system efficiently generated germ cell-deficient medaka (Table S1) and rainbow trout (*Oncorhynchus mykiss*).^22^ The simultaneous knockdown of *dnd1* and *nanos3* (DN-KD) in the present study also demonstrates promising results for host sterilization, as all non-germline chimeras became sterile (Figure 2D). In addition, transplantation of DN-OE blastomeres into a sterile host efficiently generated germline chimera and produced donor-derived gametes (Figure 2), indicating no need for germ cell isolation. Moreover, the synergistic action of *dnd1* and *nanos3* on PGC formation is likely conserved among teleost fish because *dnd1* and *nanos3* derived from phylogenetically distant zebrafish could generate iPGCs in medaka (Figures 4H and 4I). Therefore, we propose that the combination of DN-KD using the CRISPR-Cas13d system for sterile host preparation and DN-OE for donor preparation significantly enhances the efficiency of germline replacement techniques in a wide variety of fish species.

### Improvement of KI efficiency

The development of genome editing has had a tremendous impact on the fields of basic and applied research, including aquaculture.^52^ Studies on gene knockouts are constantly increasing, but site-specific gene insertion by HDR in fish remains limited.^53^ Although the efficiency of endogenous protein tagging has been greatly improved by 5′ biotinylated donor DNA^46^^,^^54^ and Cas9-mSA,^45^ tagging of endogenous GP protein has not been reported. In the conventional one-cell stage injection, we could not obtain EGFP-tagged VASA signals in medaka embryos. However, injecting KI solutions and two GPs into one blastomere of 16-cell stage embryos significantly improved the efficiency of tagging germline protein (Figure 5). This improvement can be explained by changes in cell cycle phases during embryogenesis. After fertilization, a single cell undergoes rapid and synchronous cell division without G1 and G2 phases to increase the number of blastomeres, followed by midblastula transition with zygotic genome activation accompanied by interphases.^55^ HDR is predominantly active in the S-G2 phases of the cell cycle.^56^ In medaka, asynchronous cell division and transcriptional activation are initiated at the 16-cell stage, which predominantly occurs in the center of blastomeres.^55^ Therefore, the improvement in HDR-mediated KI efficiency by 16-cell-stage injection is likely due to more frequent repair of the targeted double-stranded breaks using HDR compared to one-cell injection. A recent live imaging study of medaka embryos revealed that inner blastomeres located at the center lengthen the cell cycles earlier than those located at the periphery.^57^ The finding supports the enhanced KI efficiency by injecting the central blastomere at the 16-cell stage rather than the peripheral one (Figure S4).

### Limitations of the study

This study has pinpointed the key elements of the GP, *dnd1* and *nanos3*, that are synergistically involved in PGC formation in fish. However, the specific molecular mechanisms underlying the synergistic effects remain unexplored. In mice, there is evidence that DND1 directly interacts with NANOS3, forming a complex critical for germ cell development.^58^^,^^59^^,^^60^ Although the regulation of *nanos3* mRNA by DND1 protein has been reported in zebrafish,^33^^,^^61^ the specifics of their protein-protein interactions are still unknown in fish. Additionally, identifying the downstream targets (mRNA) of DND1 and NANOS3 is vital for an enhanced understanding of germ cell formation mechanisms. Further investigation into how DND1 and NANOS3 interact at the molecular level in fish contributes to understanding conserved and diverged mechanisms of germline development across vertebrates.

## Resource availability

### Lead contact

Further information and requests for resources and reagents should be directed to and will be fulfilled by the Lead Contact, Toshiya Nishimura (tnishi@fish.hokudai.ac.jp).

### Materials availability

Plasmids and *vasa:EGFP* knock-in transgenic medaka generated in this study are available from the lead contact upon request. This study did not generate new unique reagents.

### Data and code availability

• All data reported in this paper will be shared by the lead contact upon request.
• This paper does not report the original code.
• Any additional information required to reanalyze the data reported in this paper is available from the lead contact upon request.

## Acknowledgments

We thank NBRP Medaka for providing the medaka cDNA clones and hatching enzymes. This work was supported by 10.13039/501100001691JSPS KAKENHI (21H02277 and 24K01846 to T.N.), JSPS J-PEAKS, and JST FOREST (Fusion Oriented REsearch for disruptive Science and Technology Program) (JPMJFR210D to T.N.).

## Author contributions

Conceptualization, T.N.; methodology, T.N.; investigation, T.N.; visualization, T.N.; validation, T.N. and T.F.; writing – original draft, T.N.; writing – review and editing, T.N. and T.F.; funding acquisition, T.N.; resources, T.N.; project administration, T.N.

## Declaration of interests

Hokkaido University (T.N.) applied for a patent related to the technology described in this work. Application No. 2023–571068.

## STAR★Methods

### Key resources table

**Table:** 

REAGENT or RESOURCE	SOURCE	IDENTIFIER
**Antibodies**		

Rat monoclonal anti-GFP	Nacalai Tesque	04404-84 RRID:AB_10013361
Rabbit polyclonal anti-VASA	Aoki et al.^8^	N/A
Goat anti-rat IgG Alexa Fluor® 488	Abcam	ab150157 RRID:AB_2722511
Goat anti-rabbit IgG Alexa Fluor® 568	Abcam	ab175471 RRID:AB_2576207

**Chemicals, peptides, and recombinant proteins**		

Medaka hatching enzyme	NBRP medaka	N/A
Blocking One	Nacalai Tesque	03953-95
4′,6-diamidino-2-phenylindole (DAPI)	Invitrogen™	D1306
Ethyl 3-aminobenzoate methanesulfonate (MS-222)	Sigma-Aldrich	E10521
TURBO-DNase	Invitrogen™	AM2238
UltraPure™ DNase/RNase-Free Distilled Water	Invitrogen™	10977015
DIG RNA Labeling Mix	Roche	11277073910
T7 RNA polymerase	NEB	M0251S
KOD One® PCR Master Mix -Blue- (Dye-containing 2×PCR Master Mix)	TOYOBO	KMM-201
KOD SYBR™ qPCR Mix	TOYOBO	QKD-201
PrimeSTAR® Max DNA Polymerase	Takara Bio	R045A
NEBuilder® HiFi DNA Assembly Master Mix	NEB	E2621L
TaqMan™ Fast Advanced Master Mix	Thermo Fisher Scientific	4444557

**Critical commercial assays**		

mMESSAGE mMACHINE™ T3 Transcription Kit	Invitrogen™	AM1348
Monarch® RNA Cleanup Kit	NEB	T2040L
ScriptMAX® Thermo T7 Transcription Kit	TOYOBO	TSK-101
NucleoSpin Gel and PCR Clean-Up Kit	Takara Bio	U0609C

**Experimental models: Organisms/strains**		

Medaka (*Oryzias latipes*): OK-Cab	NBRP medaka	MT830
Medaka: *olvas*-EGFP	NBRP medaka	TG870
Medaka: *sox9b*-DsRed/*olvas*-EGFP	Nakamura et al.^36^	N/A

**Oligonucleotides**		

5' bion_M13_F: /5BiosG/GTAAAACGACGGCCAGT	IDT	N/A
5' bion_M13_R: /5BiosG/CAGGAAACAGCTATGAC	IDT	N/A
dmy-FAM, Taqman MGB probe: TGGCTTCACCGTTGGA	Thermo Fisher Scientific	N/A
cyp19a-VIC, Taqman MGB probe: ACAACAAATATGGAGACATT	Thermo Fisher Scientific	N/A
Tables S4 and S5	Sigma-Aldrich	N/A

**Recombinant DNA**		

pT3TS-RfxCas13d-HA	Kushawah et al.^24^	Addgene Plasmid #141320
pGGEV1_XcmI-LacZ	Kirchmaier et al.^62^	Addgene Plasmid #49296
pGGEV_2′_XcmI-LacZ	Kirchmaier et al.^62^	Addgene Plasmid #49303
pGGDestSC-ATG	Kirchmaier et al.^62^	Addgene Plasmid #49322
cyto-YFP-FKBPx5	Nakamura et al.^63^	Addgene Plasmid #103777
PCS2+Cas9-mSA	Gu et al.^45^	Addgene Plasmid #103882
pcs2dndChDD3′ vector	Hong et al.^12^	N/A
pGGDestSC-*nanos3*-mut-*SV40pA*	This paper	N/A
pGGDestSC-*dnd1*-mut-*SV40pA*	This paper	N/A
pGGDestSC-*nanos3*-mut-*nanos3* 3’UTR	This paper	N/A
pGGDestSC-*dnd1*-mut-*dnd1* 3’UTR	This paper	N/A
pGGDestSC-Z*nanos3*-*SV40pA*	This paper	N/A
pGGDestSC-Z*dnd1*-*SV40pA*	This paper	N/A
pGGDestSC-*FL-EGFP*	This paper	N/A
pGGDestSC*-vasa-SV40pA*	This paper	N/A
pGGDestSC*-piwil1-SV40pA*	This paper	N/A
pGGDestSC*-dazl-SV40pA*	This paper	N/A
pGGDestSC*-tdrd6-SV40pA*	This paper	N/A
pGGDestSC*-tdrd7a-SV40pA*	This paper	N/A
pGGDestSC*-dazap2-SV40pA*	This paper	N/A
pGGDestSC*-buc-SV40pA*	This paper	N/A
pGCAP10-*piwil1*	NBRP	olte25g12
pGCAP10-*tdrd1*	NBRP	olte3f09
pGCAP10-*tdrd7a*	NBRP	olte1c03
pGCAP10-*dazap2*	NBRP	olova6f16
pGEM-*buc*	This paper	N/A
pGEM-*dazl*	This paper	N/A
pGEM-*tdrd6*	This paper	N/A
pGEM-*cxcr4b*	This paper	N/A
pBlueScript-*vasa*	Tanaka et al.^29^	N/A

**Software and algorithms**		

cas13desgin tool	Wessels et al.^27^ Guo et al.^28^	https://cas13design.nygenome.org/
R	R Core Team^64^	https://www.r-project.org/
Fiji	Schindelin et al.^65^	https://imagej.net/software/fiji/

**Other**		

Thin-walled Glass Capillary Tubing	Narishige	G-100
LightCycler® 480 system II 96 well	Roche	05015278001

### Experimental model and study participant details

Medaka (*Oryzias latipes*, Cab strain) were maintained in fresh water at 26°C under a 12-hour light/12-hour dark cycle. The embryos were obtained by natural spawning from three- to six-month-old parental medaka and staged according to a previously described study.^34^ Wild-type medaka, *olvas*-EGFP single transgenic medaka,^29^ and *sox9b*-DsRed/*olvas*-EGFP double transgenic medaka^36^ were used in this study. The fish were anesthetized with ice-cold water or ethyl 3-aminobenzoate methanesulfonate (MS-222) before dissection. All animal experiments were approved by the Center for Animal Research and Education (approval #21-0063) and performed according to the guidelines of Hokkaido University.

The genotypic sex (XX-XY) of adult and larval fish was determined by real-time PCR using TaqMan MGB probes (Thermo Fisher Scientific) to detect the male-determining gene *DMY*/*dmrt1bY* and the autosomal gene *cyp19a1* (positive control). Crude genomic DNA was extracted from the larval head or the tip of the caudal fin using lysis buffer (100 mM Tris-HCl, pH 7.8; 200 mM NaCl; 0.1% SDS; 5 mM EDTA) by heating at 95°C for 10 min. PCR was performed using TaqMan™ Fast Advanced Master Mix (Thermo Fisher Scientific) on a LightCycler® 480 system II (Roche). The cycling parameters were set to 40 cycles of 95°C for 3 s and 60°C for 20 s. PCR primers used for genotyping are listed in Table S5.

### Method details

#### Knockdown screening by the CRISPR-Cas13d system

The pT3TS-RfxCas13d-HA plasmid (#141320; Addgene, Watertown, MA, USA) was a gift from Dr. Ariel A. Bazzini and Dr. Miguel A. Moreno-Mateos.^24^ The plasmid was digested with NotI, followed by *in vitro* transcription with the T3 promoter using the mMESSAGE mMACHINE™ (Invitrogen, Waltham, MA, USA). Following DNase treatment using TURBO DNase (Invitrogen), the resulting *Cas13d* mRNA was purified using a Monarch® RNA Cleanup Kit (NEB, Ipswich, MA, USA) and eluted using UltraPure™ DNase/RNase-Free Distilled Water (Invitrogen).

The full-length sequences of germline-related genes (Table S1) were uploaded using the cas13desgin tool (https://cas13design.nygenome.org/).^27^^,^^28^ We selected quartile 4 gRNAs that are known to have high knockdown efficacy.^27^^,^^28^ For each gene, three gRNAs were designed and synthesized in one tube using the ScriptMAX® Thermo T7 Transcription Kit (TOYOBO), as described previously.^22^ All primers used for gRNA synthesis are listed in Table S4.

*Cas13d* mRNA (200 ng/μl) and gRNAs (100 ng/μl) were injected into one-cell stage medaka embryos together with *EGFP-nanos3* 3′ UTR mRNA (20 ng/μl) for visualization of PGCs. The absence of EGFP-positive germ cells was examined in embryos at 4 days post-fertilization when PGCs arrived at the gonads.

#### Construction of expressing vectors for *dnd1*-SV40pA and *nanos3*-SV40pA

The protein-coding regions of medaka *nanos3* and *dnd1* were amplified using KOD One® PCR Master Mix (TOYOBO, Japan) from the cDNA of medaka ovaries and the pcs2dndChDD3′ vector (gifted by Dr. YunHan Hong and Dr. Kiyoshi Naruse),^12^ respectively. They were cloned into the BamHI and KpnI sites of pGGEV1_XcmI-LacZ (gifted by Dr. Joachim Wittbrodt, #49296; Addgene).^62^ Similarly, the protein-coding regions of zebrafish *nanos3* and *dnd1* were amplified from the cDNA of zebrafish ovaries and cloned into the BamHI and KpnI of the pGGEV_–1_XcmI-LacZ vector. *SV40pA* was amplified from the cyto-YFP-FKBPx5 vector (#103777; Addgene)^63^ and cloned into the BamHI and KpnI sites of pGGEV_2′_XcmI-LacZ (#49303; Addgene). Similarly, *nanos3* and *dnd1* 3′ UTR were amplified from the cDNA of medaka ovaries and inserted into BamHI and KpnI sites of pGGEV_2′_XcmI-LacZ. *nanos3* and *dnd1* were inserted upstream of *SV40pA* or their 3′ UTR into the pGGDestSC-ATG vector (#49322; Addgene) via Golden GATE cloning to generate pGGDestSC-*nanos3*-*SV40pA*, pGGDestSC-*dnd1*-*SV40pA,* pGGDestSC-*nanos3*-*nanos3* 3′ UTR*,* pGGDestSC-*dnd1*-*dnd1* 3′ UTR (*nanos3* and *dnd1* were derived from medaka)*,* pGGDestSC-Z*nanos3*-*SV40pA* and pGGDestSC-Z*dnd1*-*SV40pA (nanos3* and *dnd1* were derived from zebrafish) as previously described.^62^ To protect CRISPR-Cas13d mediated knockdown, medaka *dnd1* and *nanos3* gRNA-targeted sites were mutated by mutagenesis PCR according to the protocol of the PrimeSTAR Mutagenesis Basal Kit (Figures S1A and S1B, Takara Bio, Japan). The resulting vectors, pGGDestSC-*nanos3*-mut-*SV40pA,* pGGDestSC-*dnd1*-mut-*SV40pA*, pGGDestSC-*nanos3*-mut-*nanos3* 3′ UTR, and pGGDestSC-*dnd1*-mut-*dnd1* 3′ UTR were used as templates for mRNA synthesis. All primers used for the plasmid construction are listed in Table S5.

#### Construction of expressing vectors for seven germ plasm genes

The cDNA clones of *piwil1* (olte25g12), *tdrd7a* (olte1c03) and *dazap2* (olova6f16) were supplied by NBRP Medaka (https://shigen.nig.ac.jp/medaka/), while the *vasa* cDNA in pBlueScript (SK-) was a kind gift from Dr. Minoru Tanaka (Nagoya University). *dazl*, *buc*, and *tdrd6* were amplified from the cDNA of medaka ovaries and inserted into the pGEM-T Easy Vector (Promega, Madison, WI, USA) via TA-cloning. The coding regions of these genes were amplified from the cloning plasmids using KOD One® PCR Master Mix (TOYOBO, Japan) and inserted upstream of the *SV40pA* of the linearized pGGDestSC-ATG plasmid using NEBuilder® HiFi DNA Assembly Master Mix (NEB) according to the manufacturer’s instructions. All primers used for plasmid construction are listed in Table S5.

#### *In vitro* transcription

For all constructs generated using the pGGDestSC-ATG vector, DNA templates for *in vitro* transcription were amplified by PCR using the T3 promoter-tagged forward primer and the SV40pA reverse primer (Table S5), followed by purification using the NucleoSpin Gel and PCR Clean-Up Kit (Takara Bio). Using the PCR product as a template, mRNAs were synthesized using the mMESSAGE mMACHINE™ T3 Transcription Kit (Invitrogen), followed by DNase treatment using TURBO DNase (Invitrogen) and purification with the Monarch® RNA Cleanup Kit (NEB). mRNA was eluted in UltraPure™ DNase/RNase-Free Distilled Water (Invitrogen).

#### Generation of germline chimera

Germ cell-deficient hosts were generated by injection of 200 ng/μl *Cas13d* mRNA, 50 ng/μl *dnd1* gRNA2, and 50 ng/μl *nanos3* gRNA1 (Table S4) into one-cell stage embryos (non-transgenic wild-type). Donor embryos were prepared by injection of 20 ng/μl *dnd1*-*SV40pA* and 20 ng/μl *nanos3*-*SV40pA* mRNAs into one-cell stage embryos (*olvas*-EGFP/*sox9b*-DsRed transgenic line). At the blastula stage, both donor and host eggs were dechorionated by hatching enzyme (provided by NBRP Medaka), and the naked embryos were transferred into medaka balanced salt solution (BSS) supplemented with 5 mM HEPES-NaOH (pH = 7.5) and penicillin (50 U/ml)/streptomycin (50 μg/ml). Fewer than 20 or more than 50 blastomeres at the blastula stage were transplanted into host embryos at the same stage using G100 glass capillary needles (Narishige, Japan) controlled by an oil pressure manipulator (Narishige).

#### Immunohistochemical staining

Immunohistochemical staining was performed using a previously established method for detecting medaka gonads.^66^ The medaka larvae at the hatching stage and 10 day-post-hatching were fixed overnight in 4% paraformaldehyde (PFA) dissolved in phosphate-buffered saline (PBS) containing 0.1% Tween at 4°C. Subsequently, they were substituted with 100% methanol, washed with PBS + 0.1% Tween (PBST) and subjected to blocking using Blocking One (Nacalai Tesque, Japan). Anti-GFP (rat; Nacalai Tesque) and anti-VASA (rabbit) primary antibodies^8^ were used at a 400-fold dilution. Secondary antibodies, such as anti-rat IgG Alexa Fluor® 488 (Abcam, Cambridge, UK) and anti-rabbit IgG Alexa Fluor® 568 (Abcam), were used at a 400-fold dilution, and nuclei were stained with 4′,6-diamidino-2-phenylindole (DAPI). The stained samples were mounted on glass slides and observed under a confocal laser microscope (Nikon, Japan).

#### Generation of iPGCs from a specific blastomere at the 16-cell stage

At the one-cell stage, 200 ng/μl *Cas13d* mRNA, 50 ng/μl *dnd1* gRNA2*,* 50 ng/μl *nanos3* gRNA1, and 20 ng/μl *mCherry*-*nanos3* 3′ UTR mRNA were injected to deplete endogenous *dnd1* and *nanos3*. At the 16-cell stage, 10 ng/μl *dnd1-*mut*-SV40pA* and *nanos3-*mut*-SV40pA* mRNA were injected into one blastomere located at either the corner or the center. For the injection of mRNAs for nine GP (*vasa*, *dazl*, *piwil1*, *dnd1*, *nanos3*, *tdrd6*, *tdrd7a*, *dazap2*, and *buc*) and eight GP (without *dnd1* or *nanos3*), 10 ng/μl of each mRNA was injected into one blastomere at the corner of the 16-cell stage embryos.

#### Generation of *vasa:EGFP* knock-in medaka

The flexible linker (*FL*)^67^ and *EGFP* were connected by Golden GATE cloning to generate *FL-EGFP*. Subsequently, the construct was amplified using primers containing M13 primer sequences and a 40 nt short homology arm of *vasa* (Figure S3A). Using the first PCR product as templates, 5′ biotinylated donor DNA was amplified by PCR using biotinylated M13 primers (IDT, Coralville, IA, USA) (Figure S3A and Table S5), followed by purification using the NucleoSpin Gel and PCR Clean-Up Kit (Takara Bio).

Cas9 conjugated with monomeric Streptavidin (PCS2+Cas9-mSA) was a gift from Dr. Janet Rossant (#103882; Addgene).^45^ The DNA template for the *in vitro* transcription of *Cas9-mSA* mRNA was prepared by PCR using a T3 promoter-tagged forward primer and SV40pA reverse primers (Table S5), followed by *in vitro* transcription as described above (***in vitro* transcription).** *vasa-*gRNA was generated as previously described (Table S5).^68^ The procedures for blastomere injection at the 16-cell stage were the same as described above (generation of iPGCs from a specific blastomere at the 16-cell stage). At the 16-cell stage, in addition to 10 ng/μl *dnd1*-mut-*SV40pA* and *nanos3-*mut-*SV40pA* mRNA, 50 ng/μl *Cas9-mSA* mRNA, 10 ng/μl *vasa*-gRNA, and 5 ng/μl 5′ biotinylated donor DNA were co-injected. The injected embryos were kept in a 29°C incubator until hatching, followed by being raised in a fish tank at 26°C.

#### Quantitative reverse transcription (q-RT)-PCR

To compare the stability of *dnd1*/*nanos3* mRNA with *SV40pA* and their 3′ UTRs, we quantified the expression levels of injected mRNA by q-RT-PCR. To equalize the molarity between the mRNA with *SV40pA* and the original 3′ UTR, we adjusted the concentrations as follows: 17 ng/μl *nanos3*-mut-*nanos3* 3′ UTR (776 nt, MW = 480 kD), 20 ng/μl *nanos3*-mut-*SV40pA* (897 nt, MW = 554 kD), 30 ng/μl *dnd1*-mut-*dnd1* 3′ UTR (2126 nt, MW = 1310 kD), and 20 ng/μl *dnd1*-mut-*SV40pA* (1410 nt, MW = 871 kD). We injected *dnd1* and *nanos3* mRNA with *SV40pA* or their 3′ UTR into one-cell stage embryos, along with *EGFP-SV40pA* for normalization. Total RNA was extracted from 15 embryos at the late blastula stage (stage 10) per sample, followed by reverse transcription with 100 ng of total RNA using the ReverTra Ace qPCR RT kit (TOYOBO). PCR reaction and quantification were performed using KOD SYBR™ qPCR mix and LightCycler® 480 system II (Roche). The analyses were conducted with two technical replicates per sample, using a total of five independent biological replicates (n = 5). The primers used for q-RT-PCR are shown in Table S5. The forward primers for *dnd1* and *nanos3* were designed on the mutation sites of the injected mRNAs (Figures S1A and S1B), thereby enabling the quantification of the injected mRNAs that can be distinguished from the endogenous transcripts.

#### *In situ* hybridization

Whole-mount *in situ* hybridization was performed using medaka embryos at stage 29 as previously described.^44^^,^^69^ DNA templates (*piwil1*, *tdrd1*, *cxcr4b*, and *dazl*) for RNA-DIG labeling by *in vitro* transcription were produced by PCR amplification using gene-specific forward and T7-tagged reverse primers (Table S5), followed by purification using the NucleoSpin Gel and PCR Clean-Up Kit (Takara Bio). The DNA template for the *vasa* probe was prepared by linearizing the plasmid by NotI digestion. RNA-DIG probes were synthesized using DIG RNA Labeling Mix (Roche, Switzerland) and T7 RNA polymerase (NEB), followed by DNase treatment using TURBO DNase (Invitrogen) and purification with the Monarch® RNA Cleanup Kit (NEB).

### Quantification and statistical analysis

#### Image analyses for estimation of iPGCs number

Due to the difficulty in counting the numerous iPGCs, we estimated the number by measuring the area of EGFP-positive iPGCs. Images of medaka embryos with *olvas*-EGFP-labeled iPGCs at stage 29 were obtained through an EGFP bandpass filter using DS-Fi3 digital camera (Nikon) on an SMZ18 stereomicroscope (Nikon). The RGB images (2880 x 2048 pixels) were converted into 8-bit images, followed by background subtraction (Rolling ball radius: 100 pixel) using Fiji.^65^ The EGFP-positive area was segmented using the MaxEntropy thresholding function and measured by the analyze particle function (size pixelˆ2: 10-Infinity) using Fiji. The average number and area (pixel) of control PGCs in embryos at stage 29 (n = 29) were 20.4 and 8629, respectively, and the area of one PGC was calculated as 423 pixels. Thus, the number of iPGCs was calculated as the EGFP-positive area (pixel) divided by 423.

#### Statistical analyses

The statistical analyses used to evaluate the data are specified in the figure legends, accompanied by the sample size (n). Statistical analyses (Welch’s t-test, Fisher's exact test, Kruskal-Wallis and Dunn's test) were performed using R software.^64^ Statistical significance was defined as p < 0.01.

## References

[1] Extavour C.G., Akam M. (2003). Mechanisms of germ cell specification across the metazoans: epigenesis and preformation. Development.

[2] Irie N., Tang W.W.C., Azim Surani M. (2014). Germ cell specification and pluripotency in mammals: a perspective from early embryogenesis. Reprod. Med. Biol..

[3] Hayashi K., Ohta H., Kurimoto K., Aramaki S., Saitou M. (2011). Reconstitution of the Mouse Germ Cell Specification Pathway in Culture by Pluripotent Stem Cells. Cell.

[4] Hayashi K., Ogushi S., Kurimoto K., Shimamoto S., Ohta H., Saitou M. (2012). Offspring from Oocytes Derived from in Vitro Primordial Germ Cell–like Cells in Mice. Science.

[5] Olsen L.C., Aasland R., Fjose A. (1997). A *vasa-like* gene in zebrafish identifies putative primordial germ cells. Mech. Dev..

[6] Houwing S., Kamminga L.M., Berezikov E., Cronembold D., Girard A., van den Elst H., Filippov D.V., Blaser H., Raz E., Moens C.B. (2007). A Role for Piwi and piRNAs in Germ Cell Maintenance and Transposon Silencing in Zebrafish. Cell.

[7] Zhao H., Duan J., Cheng N., Nagahama Y. (2012). Specific expression of *Olpiwi1* and *Olpiwi2* in medaka (*Oryzias latipes*) germ cells. Biochem. Biophys. Res. Commun..

[8] Aoki Y., Nagao I., Saito D., Ebe Y., Kinjo M., Tanaka M. (2008). Temporal and spatial localization of three germline-specific proteins in medaka. Dev. Dyn..

[9] Xu H., Li M., Gui J., Hong Y. (2007). Cloning and expression of medaka *dazl* during embryogenesis and gametogenesis. Gene Expr. Patterns.

[10] Li M., Zhu F., Li Z., Hong N., Hong Y. (2016). Dazl is a critical player for primordial germ cell formation in medaka. Sci. Rep..

[11] Weidinger G., Stebler J., Slanchev K., Dumstrei K., Wise C., Lovell-Badge R., Thisse C., Thisse B., Raz E. (2003). *dead end*, a Novel Vertebrate Germ Plasm Component, Is Required for Zebrafish Primordial Germ Cell Migration and Survival. Curr. Biol..

[12] Hong N., Li M., Yuan Y., Wang T., Yi M., Xu H., Zeng H., Song J., Hong Y. (2016). Dnd Is a Critical Specifier of Primordial Germ Cells in the Medaka Fish. Stem Cell Rep..

[13] Köprunner M., Thisse C., Thisse B., Raz E. (2001). A zebrafish *nanos*-related gene is essential for the development of primordial germ cells. Genes Dev..

[14] Aoki Y., Nakamura S., Ishikawa Y., Tanaka M. (2009). Expression and syntenic analyses of four *nanos* genes in medaka. Zoolog. Sci..

[15] Bontems F., Stein A., Marlow F., Lyautey J., Gupta T., Mullins M.C., Dosch R. (2009). Bucky Ball Organizes Germ Plasm Assembly in Zebrafish. Curr. Biol..

[16] Song P., Sun B., Zhu Y., Zhong Y., Guo J., Gui L., Li M. (2021). Bucky ball induces primordial germ cell increase in medaka. Gene.

[17] Gross-Thebing T., Yigit S., Pfeiffer J., Reichman-Fried M., Bandemer J., Ruckert C., Rathmer C., Goudarzi M., Stehling M., Tarbashevich K. (2017). The Vertebrate Protein Dead End Maintains Primordial Germ Cell Fate by Inhibiting Somatic Differentiation. Dev. Cell.

[18] Wang X., Zhu J., Wang H., Deng W., Jiao S., Wang Y., He M., Zhang F., Liu T., Hao Y. (2023). Induced formation of primordial germ cells from zebrafish blastomeres by germplasm factors. Nat. Commun..

[19] Kirchmaier S., Naruse K., Wittbrodt J., Loosli F. (2015). The Genomic and Genetic Toolbox of the Teleost Medaka (*Oryzias latipes*). Genetics.

[20] Matsuda M., Nagahama Y., Shinomiya A., Sato T., Matsuda C., Kobayashi T., Morrey C.E., Shibata N., Asakawa S., Shimizu N. (2002). *DMY* is a Y-specific DM-domain gene required for male development in the medaka fish. Nature.

[21] Nanda I., Kondo M., Hornung U., Asakawa S., Winkler C., Shimizu A., Shan Z., Haaf T., Shimizu N., Shima A. (2002). A duplicated copy of *DMRT1* in the sex-determining region of the Y chromosome of the medaka, *Oryzias latipes*. Proc. Natl. Acad. Sci. USA.

[22] Nishimura T., Takahashi E., Fujimoto T. (2024). Sterilization of fish through adaptable gRNAs targeting *dnd1* using CRISPR-Cas13d system. Aquaculture.

[23] Konermann S., Lotfy P., Brideau N.J., Oki J., Shokhirev M.N., Hsu P.D. (2018). Transcriptome Engineering with RNA-Targeting Type VI-D CRISPR Effectors. Cell.

[24] Kushawah G., Hernandez-Huertas L., Abugattas-Nuñez del Prado J., Martinez-Morales J.R., DeVore M.L., Hassan H., Moreno-Sanchez I., Tomas-Gallardo L., Diaz-Moscoso A., Monges D.E. (2020). CRISPR-Cas13d Induces Efficient mRNA Knockdown in Animal Embryos. Dev. Cell.

[25] Skvortsova K., Tarbashevich K., Stehling M., Lister R., Irimia M., Raz E., Bogdanovic O. (2019). Retention of paternal DNA methylome in the developing zebrafish germline. Nat. Commun..

[26] Shi D.-L. (2024). Interplay of RNA-binding proteins controls germ cell development in zebrafish. J. Genet. Genomics.

[27] Wessels H.-H., Méndez-Mancilla A., Guo X., Legut M., Daniloski Z., Sanjana N.E. (2020). Massively parallel Cas13 screens reveal principles for guide RNA design. Nat. Biotechnol..

[28] Guo X., Rahman J.A., Wessels H.-H., Méndez-Mancilla A., Haro D., Chen X., Sanjana N.E. (2021). Transcriptome-wide Cas13 guide RNA design for model organisms and viral RNA pathogens. Cell Genom..

[29] Tanaka M., Kinoshita M., Kobayashi D., Nagahama Y. (2001). Establishment of medaka (*Oryzias latipes*) transgenic lines with the expression of green fluorescent protein fluorescence exclusively in germ cells: a useful model to monitor germ cells in a live vertebrate. Proc. Natl. Acad. Sci. USA.

[30] Takeda Y., Mishima Y., Fujiwara T., Sakamoto H., Inoue K. (2009). DAZL Relieves miRNA-Mediated Repression of Germline mRNAs by Controlling Poly(A) Tail Length in Zebrafish. PLoS One.

[31] Škugor A., Slanchev K., Torgersen J.S., Tveiten H., Andersen Ø. (2014). Conserved Mechanisms for Germ Cell-Specific Localization of *nanos3* Transcripts in Teleost Species with Aquaculture Significance. Mar. Biotechol..

[32] Mishima Y., Giraldez A.J., Takeda Y., Fujiwara T., Sakamoto H., Schier A.F., Inoue K. (2006). Differential Regulation of Germline mRNAs in Soma and Germ Cells by Zebrafish miR-430. Curr. Biol..

[33] Kedde M., Strasser M.J., Boldajipour B., Oude Vrielink J.A.F., Slanchev K., le Sage C., Nagel R., Voorhoeve P.M., van Duijse J., Ørom U.A. (2007). RNA-Binding Protein Dnd1 Inhibits MicroRNA Access to Target mRNA. Cell.

[34] Iwamatsu T. (2004). Stages of normal development in the medaka *Oryzias latipes*. Mech. Dev..

[35] Kurokawa H., Aoki Y., Nakamura S., Ebe Y., Kobayashi D., Tanaka M. (2006). Time-lapse analysis reveals different modes of primordial germ cell migration in the medaka *Oryzias latipes*. Dev. Growth Differ..

[36] Nakamura S., Kobayashi K., Nishimura T., Higashijima S.I., Tanaka M. (2010). Identification of germline stem cells in the ovary of the teleost medaka. Science.

[37] Kurokawa H., Saito D., Nakamura S., Katoh-Fukui Y., Ohta K., Baba T., Morohashi K.I., Tanaka M. (2007). Germ cells are essential for sexual dimorphism in the medaka gonad. Proc. Natl. Acad. Sci. USA.

[38] Morinaga C., Saito D., Nakamura S., Sasaki T., Asakawa S., Shimizu N., Mitani H., Furutani-Seiki M., Tanaka M., Kondoh H. (2007). The *hotei* mutation of medaka in the anti-Mullerian hormone receptor causes the dysregulation of germ cell and sexual development. Proc. Natl. Acad. Sci. USA.

[39] Nakamura S., Watakabe I., Nishimura T., Picard J.Y., Toyoda A., Taniguchi Y., di Clemente N., Tanaka M. (2012). Hyperproliferation of mitotically active germ cells due to defective anti-Mullerian hormone signaling mediates sex reversal in medaka. Development.

[40] Nakamura S., Watakabe I., Nishimura T., Toyoda A., Taniguchi Y., Tanaka M. (2012). Analysis of medaka *sox9* orthologue reveals a conserved role in germ cell maintenance. PLoS One.

[41] Nishimura T., Tanaka M. (2014). Gonadal development in fish. Sex. Dev..

[42] Saito D., Morinaga C., Aoki Y., Nakamura S., Mitani H., Furutani-Seiki M., Kondoh H., Tanaka M. (2007). Proliferation of germ cells during gonadal sex differentiation in medaka: Insights from germ cell-depleted mutant *zenzai*. Dev. Biol..

[43] Gill M.E., Hu Y.-C., Lin Y., Page D.C. (2011). Licensing of gametogenesis, dependent on RNA binding protein DAZL, as a gateway to sexual differentiation of fetal germ cells. Proc. Natl. Acad. Sci. USA.

[44] Nishimura T., Yamada K., Fujimori C., Kikuchi M., Kawasaki T., Siegfried K.R., Sakai N., Tanaka M. (2018). Germ cells in the teleost fish medaka have an inherent feminizing effect. PLoS Genet..

[45] Gu B., Posfai E., Rossant J. (2018). Efficient generation of targeted large insertions by microinjection into two-cell-stage mouse embryos. Nat. Biotechnol..

[46] Seleit A., Aulehla A., Paix A. (2021). Endogenous protein tagging in medaka using a simplified CRISPR/Cas9 knock-in approach. Elife.

[47] Dranow D.B., Tucker R.P., Draper B.W. (2013). Germ cells are required to maintain a stable sexual phenotype in adult zebrafish. Dev. Biol..

[48] Zhang F., Li X., He M., Ye D., Xiong F., Amin G., Zhu Z., Sun Y. (2020). Efficient generation of zebrafish maternal-zygotic mutants through transplantation of ectopically induced and Cas9/gRNA targeted primordial germ cells. J. Genet. Genomics.

[49] Shimada A., Yabusaki M., Niwa H., Yokoi H., Hatta K., Kobayashi D., Takeda H. (2008). Maternal-zygotic medaka mutants for *fgfr1* reveal its essential role in the migration of the axial mesoderm but not the lateral mesoderm. Development.

[50] Yoshizaki G., Yazawa R. (2019). Application of surrogate broodstock technology in aquaculture. Fish. Sci..

[51] Goto R., Saito T. (2019). A state-of-the-art review of surrogate propagation in fish. Theriogenology.

[52] Yang Z., Yu Y., Tay Y.X., Yue G.H. (2022). Genome editing and its applications in genetic improvement in aquaculture. Rev. Aquaculture.

[53] Albadri S., Del Bene F., Revenu C. (2017). Genome editing using CRISPR/Cas9-based knock-in approaches in zebrafish. Methods.

[54] Gutierrez-Triana J.A., Tavhelidse T., Thumberger T., Thomas I., Wittbrodt B., Kellner T., Anlas K., Tsingos E., Wittbrodt J. (2018). Efficient single-copy HDR by 5’ modified long dsDNA donors. Elife.

[55] Kraeussling M., Wagner T.U., Schartl M. (2011). Highly Asynchronous and Asymmetric Cleavage Divisions Accompany Early Transcriptional Activity in Pre-Blastula Medaka Embryos. PLoS One.

[56] Hustedt N., Durocher D. (2016). The control of DNA repair by the cell cycle. Nat. Cell Biol..

[57] Kiyomitsu A., Nishimura T., Hwang S.J., Ansai S., Kanemaki M.T., Tanaka M., Kiyomitsu T. (2024). Ran-GTP assembles a specialized spindle structure for accurate chromosome segregation in medaka early embryos. Nat. Commun..

[58] Suzuki A., Niimi Y., Shinmyozu K., Zhou Z., Kiso M., Saga Y. (2016). Dead end1 is an essential partner of NANOS2 for selective binding of target RNAs in male germ cell development. EMBO Rep..

[59] Niimi Y., Imai A., Nishimura H., Yui K., Kikuchi A., Koike H., Saga Y., Suzuki A. (2019). Essential role of mouse Dead end1 in the maintenance of spermatogonia. Dev. Biol..

[60] Imai A., Hagiwara Y., Niimi Y., Tokumoto T., Saga Y., Suzuki A. (2020). Mouse dead end1 acts with *Nanos2* and *Nanos3* to regulate testicular teratoma incidence. PLoS One.

[61] Westerich K.J., Tarbashevich K., Schick J., Gupta A., Zhu M., Hull K., Romo D., Zeuschner D., Goudarzi M., Gross-Thebing T., Raz E. (2023). Spatial organization and function of RNA molecules within phase-separated condensates in zebrafish are controlled by Dnd1. Dev. Cell.

[62] Kirchmaier S., Lust K., Wittbrodt J. (2013). Golden GATEway Cloning – A Combinatorial Approach to Generate Fusion and Recombination Constructs. PLoS One.

[63] Nakamura H., Lee A.A., Afshar A.S., Watanabe S., Rho E., Razavi S., Suarez A., Lin Y.-C., Tanigawa M., Huang B. (2018). Intracellular production of hydrogels and synthetic RNA granules by multivalent molecular interactions. Nat. Mater..

[64] R Core Team (2023). R: A Language and Environment for Statistical Computing. R Foundation for Statistical Computing. (Vienna, Austria).

[65] Schindelin J., Arganda-Carreras I., Frise E., Kaynig V., Longair M., Pietzsch T., Preibisch S., Rueden C., Saalfeld S., Schmid B. (2012). Fiji: an open-source platform for biological-image analysis. Nat. Methods.

[66] Nishimura T., Tanaka M., Dosch R. (2021). Germline Development in the Zebrafish: Methods and Protocols.

[67] Gutierrez-Triana J.A., Mateo J.L., Ibberson D., Ryu S., Wittbrodt J. (2016). iDamIDseq and iDEAR: an improved method and computational pipeline to profile chromatin-binding proteins. Development.

[68] Kikuchi M., Nishimura T., Ishishita S., Matsuda Y., Tanaka M. (2020). *foxl3*, a sexual switch in germ cells, initiates two independent molecular pathways for commitment to oogenesis in medaka. Proc. Natl. Acad. Sci. USA.

[69] Nakamura S., Kobayashi D., Aoki Y., Yokoi H., Ebe Y., Wittbrodt J., Tanaka M. (2006). Identification and lineage tracing of two populations of somatic gonadal precursors in medaka embryos. Dev. Biol..

