# Supplementary material for: Generation of primordial germ cell-like cells by two germ plasm components, dnd1 and nanos3, in medaka (Oryzias latipes)
Source: iScience. 2025 Feb 8;28(3):111977. doi: 10.1016/j.isci.2025.111977 (PMC11889698; doi:10.1016/j.isci.2025.111977)
Supplement: Document S1. Figures S1–S5 and Tables S1–S5 [file mmc1.pdf]

iScience, Volume *28*

Supplemental information

Generation of primordial germ cell-like cells by two germ plasm components, *dnd1* and *nanos3*, in medaka (*Oryzias latipes*)

Toshiya Nishimura and Takafumi Fujimoto

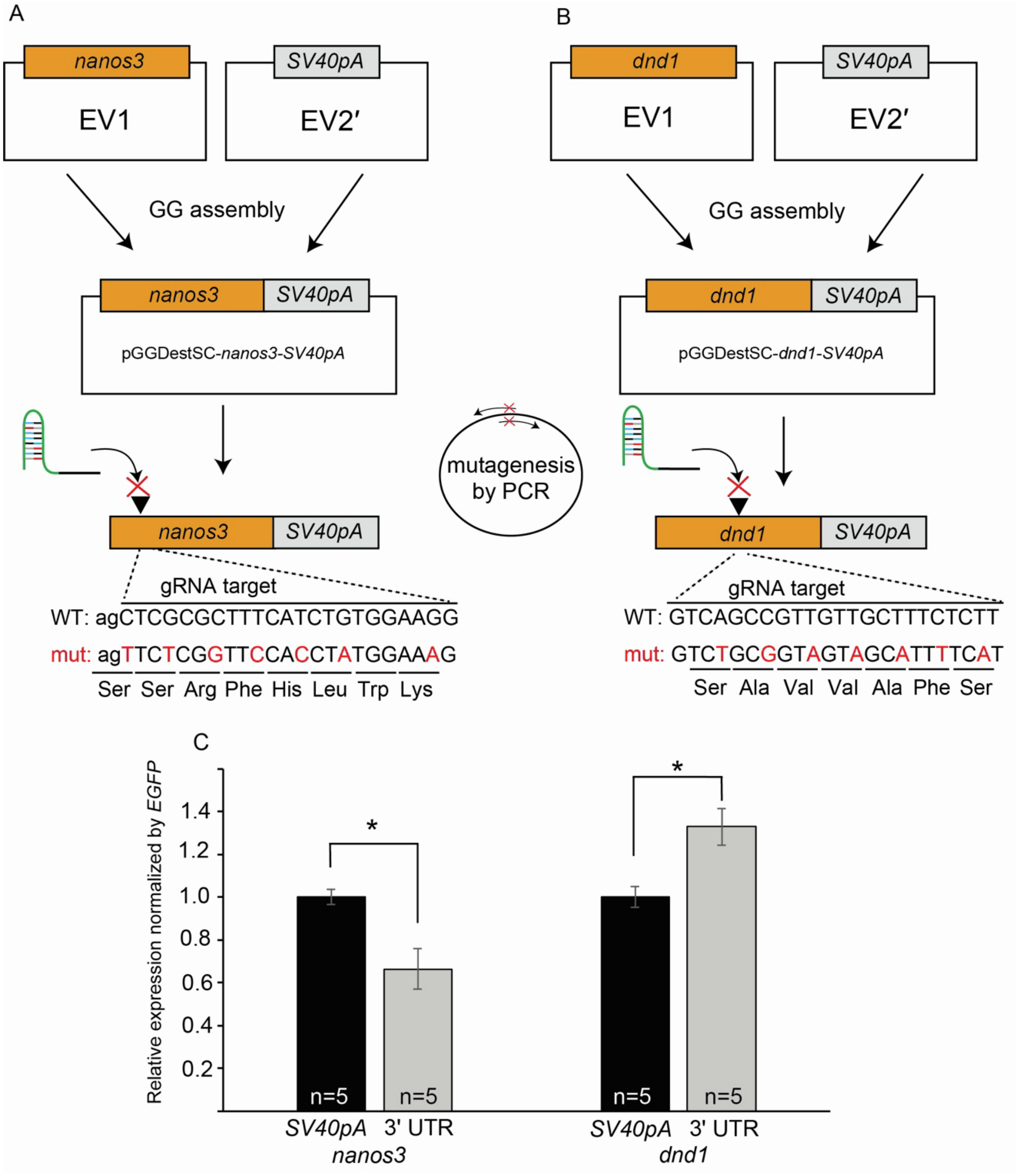

Figure S1. Construction of *nanos3-SV40pA* and *dnd1-SV40pA* vectors, related to Figure 1, 2, 4 and STAR methods. (A) Construction of the *nanos3-SV40pA* vector. The *nanos3* coding region and *SV40pA* were inserted into entry vector 1 (EV1) and EV2′. Subsequently, these components were assembled using Golden GATE (GG) assembly to generate the pGGDestSC-*nanos3-SV40pA*. (B) Construction of the *dnd1-SV40pA* vector. The *dnd1* coding region and *SV40pA* were similarly inserted into EV1 and EV2′, followed by GG assembly to generate the pGGDestSC-*dnd1-SV40pA*. Silent mutations (red nucleotides) were introduced into the gRNA

target sites of *nanos3* and *dnd1* via mutagenesis PCR. (C) Comparison of stability between *nanos3*/*dnd1* with *SV40pA* and *nanos3*/*dnd1* with their 3′ UTR (*nanos3*/*dnd1*-3′ UTR) by quantitative (q)-PCR. *nanos3*/*dnd1*-*SV40pA* or *nanos3*/*dnd1*-3′ UTR mRNA was injected together with *EGFP*-*SV40pA* into the one-cell stage, followed by q-PCR analyses at the blastula stage. The relative expression of each transcript normalized by *EGFP* was calculated by the ΔΔCt method. Whereas *nanos3*-3′ UTR was less stable than *nanos3*-*SV40pA* mRNA, *dnd1*-3′ UTR was more stable than *dnd1*-*SV40pA.* Asterisks indicate statistical significance by Welch's t-test ($p < 0.01$). The error bars in the graph represent the standard deviation.

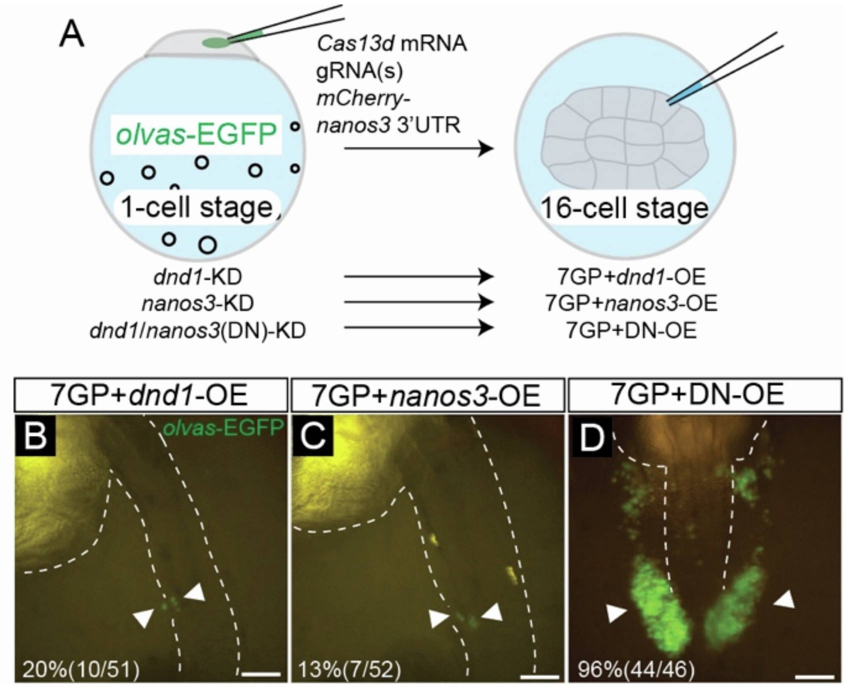

Figure S2. Generation of iPGCs with nine germ plasm (GP) genes, related to Figure 4. (A) Schematic representation of iPGC generation from a specific blastomere. At the one-cell stage of *olvas*-EGFP transgenic medaka, knockdown (KD) of *dnd1*, *nanos3*, or a combination of *dnd1* and *nanos3* (DN) was performed using the CRISPR-Cas13d system. At the 16-cell stage, 7 GP genes (*vasa*, *dazl*, *piwil1*, *tdrd6*, *tdrd7a*, *dazap2*, and *buc*) with *dnd1* (7GP + *dnd1*), *nanos3* (7GP + *nanos3*), *or both dnd1 and nanos3* (7GP + DN) mRNA were injected into the corner of a blastomere for overexpression (OE). (B–C) Injection of 7GP + *dnd1* or *nanos3* mRNA into *dnd1* or *nanos3*-KD embryos resulted in the minimal recovery of *olvas*-EGFP germ cells (arrowheads) in the stage 29 embryos. The "%" indicates the rate of embryos with at least one EGFP-positive germ cell. (D) Injection of 7GP + DN mRNAs resulted in the generation of large germ cell clusters (arrowheads). The "%" indicates the rate of embryos with large germ cell clusters. Embryonic bodies are outlined by white dotted lines (B–D). Scale bars: 100 μm.

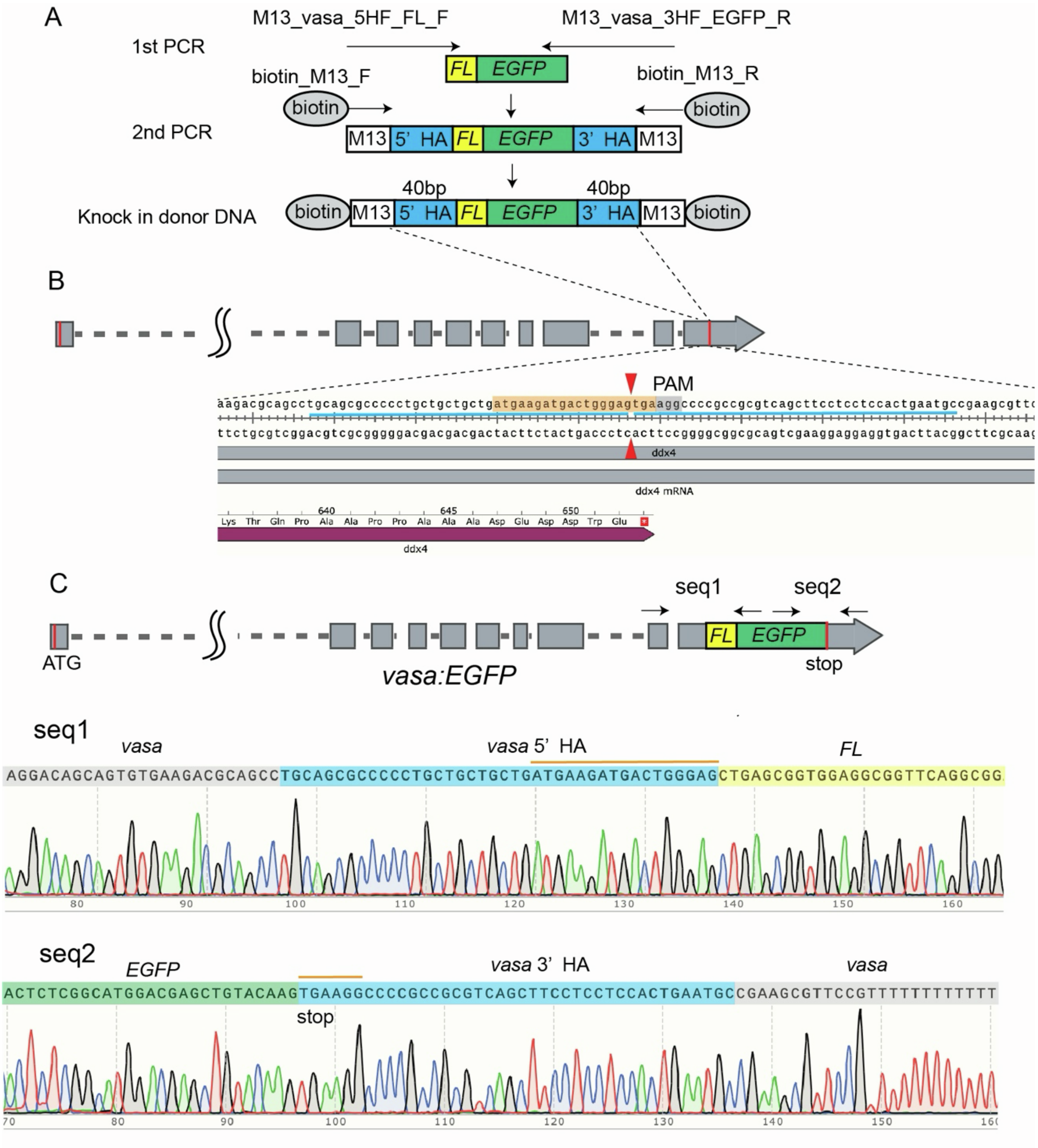

Figure S3. Generation of *vasa:EGFP* knock-in medaka, related to Figure 5 and STAR methods. (A) Schematic representation of PCR-based donor DNA synthesis. In the first PCR, a flexible linker (FL)-EGFP construct flanked by 40 bp *vasa* 5′ and 3′ homology arms (HA) with M13 sequences was generated by using forward and reverse primers specific to the FL and EGFP. In the second PCR, 5′ biotinylated donor DNA was synthesized using 5′ biotinylated M13 forward and reverse primers. (B) The 5′ biotinylated donor DNA was integrated just upstream of the *vasa* stop codon (TGA) using *vasa*-gRNA and Cas9-mSA. Red arrowheads indicate the double-strand break site created by Cas9. The *vasa*-gRNA target region is highlighted in orange. The sequences of homology arms are underlined in blue. (C) Schematic representation of the

resultant *vasa:EGFP* knock-in allele. The position of primer sets used for sequence analysis (seq1 and seq2) are indicated by arrows. Orange lines above the nucleotide sequence highlighted blue indicate the *vasa*-gRNA target region.

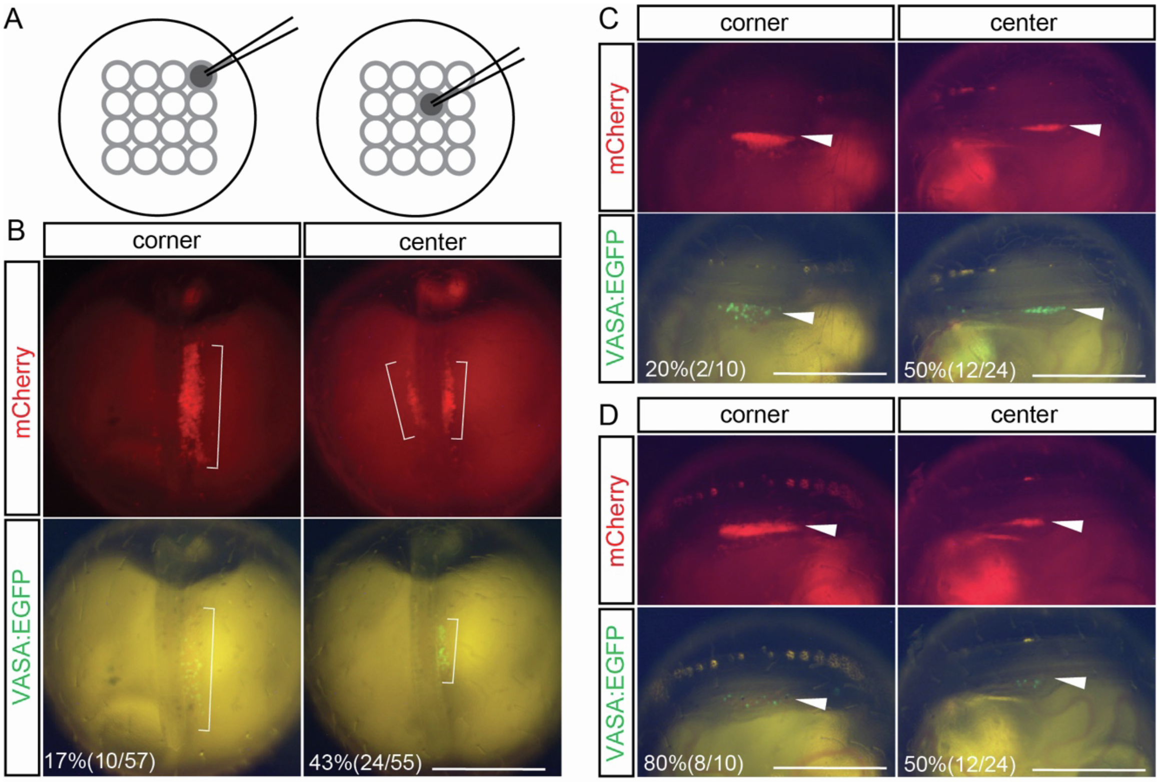

Figure S4. Comparison of knock-in efficiency between corner and center of blastomere injection at the 16-cell stage, related to Figure 5. (A) The position of the corner or center of the blastomere at the 16-cell stage for knock-in (KI) injection. KI injectates and *dnd1*/*nanos3* mRNAs were injected into either the corner or center of the blastomere at the 16-cell stage. (B) Medaka embryos at stage 22 (somite stage). iPGCs are visualized with mCherry (red), and iPGCs with *EGFP* integration into the *vasa* locus are shown in green (VASA:EGFP). The square brackets indicate the position of iPGCs. The "%" indicates the rate of VASA:EGFP-positive embryos. (C) Medaka embryos at stage 35, where most VASA:EGFP-positive cells (green) overlap with iPGCs (red). (D) Medaka embryos at stage 35 showing VASA:EGFP-positive cells sparsely distributed in iPGCs. Arrowheads indicate the position of gonads. The "%" indicates the rate of VASA:EGFP-positive embryos. Scale bars: 500 μm.

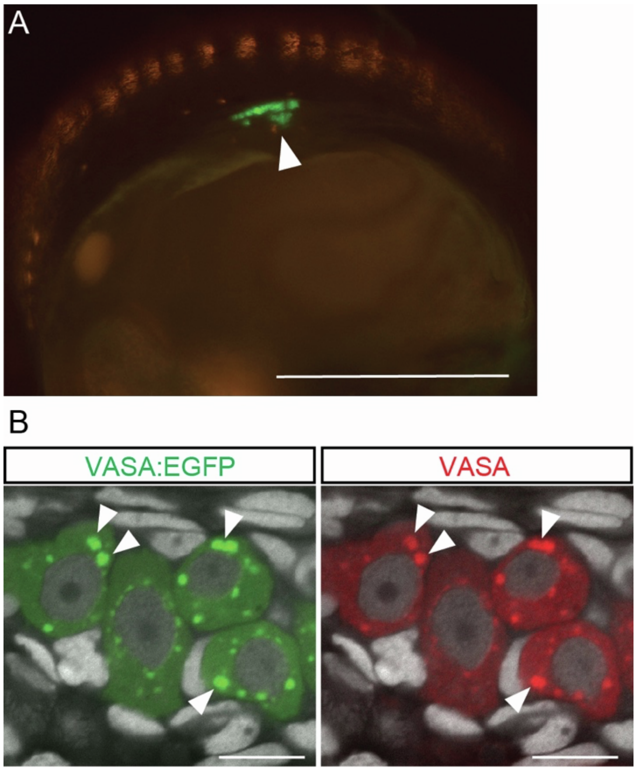

Figure S5. Stable transmission of *vasa*:*EGFP* knock-in allele, related to Figure 5. (A) The fourth generation (F4) of *vasa*:*EGFP* medaka embryos at stage 35. The arrowhead indicates VASA:EGFP-positive germ cells (green). (B) Immunohistochemical staining of germ cells in *vasa*:*EGFP* embryos at the hatching stage. VASA:EGFP (green) and endogenous VASA (red) were colocalized (arrowheads). Nuclei were stained with DAPI (gray). Scale bars: 500 μm in A and 10 μm in B.

Table S1. Knockdown (KD) screening of germ plasm and germ cell-related genes by CRISPR-Cas13d system, related to Figure 1, Table S4, and STAR Methods

KD gene	reference gene ID	# of injected eggs	# of dead or abnormal embryos	# of embryos checked for germless	# of germless embryos	% of germless embryos*
Cas13d only	–	45	8	37	0	0%
dnd1	NM_001164516	30	9	21	21	100%
nanos3	NM_001122828	34	4	30	29	97%
tdrd1	AB306932	14	0	14	0	0%
tdrd5	XM_011474039	13	2	11	0	0%
tdrd6	NM_001360941	14	10	4	0	0%
tdrd7a	XM_011480821	14	2	12	0	0%
tdrd7b	XM_004079730	12	1	11	0	0%
tdrd9	XM_023951948	17	5	12	1	8%
tdrd12	XM_004086241	14	0	14	0	0%
ddx4	NM_001104676	35	1	34	1	3%
ddx19	XM_004070896	16	1	15	1	7%
dazl	NM_001104799	33	3	30	2	7%
piwil1	NM_001160436	17	2	15	0	0%
piwil2	NM_001160437	18	4	14	0	0%
mov10L	XM_004069901	17	1	16	0	0%
buc	XM_004081355	17	1	16	0	0%
bucL	XM_011489116	19	2	17	0	0%
elavl2	XM_011487340	19	0	19	0	0%
gdf3	XM_004081435	15	9	6	0	0%
gpat2	XM_011478919	16	4	12	0	0%
gasz	XM_004086224	16	5	11	0	0%
golga3	XM_011479513	18	5	13	0	0%
rbm46	XM_011487179	16	1	15	0	0%
rnf17	XM_023950859	10	1	9	1	11%
rnf213a	XM_011478164	20	5	15	0	0%
rnf213b	XM_020712049	19	0	19	0	0%
smc1b	XM_020703649	14	0	14	0	0%
taf4b	XM_004067653	14	2	12	0	0%
topaz	XM_023959893	15	0	15	0	0%
adad1	olte38g04	14	0	14	0	0%

* The % of germless embryos was calculated as (number of germless embryos) / (number of embryos checked).

Table S2. Fertility, sex reversal (SXR), and germ cell deficiency in 3-month-old adult germline chimeras, related to Figures 2 and 3

host	donor	# of chimera	XX♀	XY♂	XX♂ (SXR)	% of SXR	% of fertility	% of germless
DN-KD*	control BM: < 20	19	7	10	2	22%	0%	100%
DN-KD	DN-OE BM**: < 20	19	11	8	0	0%	74%	16%
DN-KD	control BM: > 50	16	8	7	1	11%	88%	6%
DN-KD	DN-OE BM: > 50	17	6	6	5	45%	94%	6%
DN-KD	DNZ-OE BM***: > 50	27	16	7	4	25%	96%	0%
control	–	18	9	9	0	0%	100%	0%

* DN-KD: Sterilization by knockdown of *dnd1* and *nanos3* using the CRISPR-Cas13d system.

** DN-OE BM: *dnd1* and *nanos3* overexpressed blastomeres.

*** DNZ-OE BM: *dnd1, nanos3*, and *dazl* overexpressed blastomeres.

Table S3. Fertility, sex reversal (SXR), and germ cell deficiency in 3-month-old adult fish derived from embryos with iPGCs, related to Figure 4

sample	# of fish	XX♀	XY♂	XX♂ (SXR)	% of SXR	% of fertility	% of germless
control	46	21	25	0	0%	100%	0%
DN-OE*	48	11	26	11	50%	96%	0%
Z-DN-OE**	32	6	18	8	57%	94%	3%

* DN-OE: Injection of medaka *dnd1* and *nanos3* mRNA into one blastomere of medaka embryos at the16-cell stage.

** Z-DN-OE: Injection of zebrafish *dnd1* and *nanos3* mRNA into one blastomere of medaka embryos at the16-cell stage.

Table S4. Primers used for gRNA synthesis in the CRISPR-Cas13d system, related to STAR Methods

primer name	5'-sequence-3'	Note
universal-F+13nt	GGCCAGTGAATTGTAATACGACTCACTATAGGAACCCCTACCAACTGGTCGGGGTTTGAAAC	KD screening, generation of germless host
dnd1_gRNA1	gcgcgctgtgaggtctttatcagGTTTCAAACCCCGACCAGTT	KD screening
dnd1_gRNA2	gtcagccgttgttgctttctcttGTTTCAAACCCCGACCAGTT	KD screening, generation of germless host
dnd1_gRNA3	gggacaagtcatgttttctgtgtGTTTCAAACCCCGACCAGTT	KD screening
nanos3_gRNA1	ctcgcgctttcatctgtggaaggGTTTCAAACCCCGACCAGTT	KD screening, generation of germless host
nanos3_gRNA2	ggagtctggacttcttcatctgaGTTTCAAACCCCGACCAGTT	KD screening
nanos3_gRNA3	aaggtggacagaacttattcttcGTTTCAAACCCCGACCAGTT	KD screening
tdrd1_gRNA1	atggatggcagtacatcttttggGTTTCAAACCCCGACCAGTT	KD screening
tdrd1_gRNA2	tttggctgctgatcaaaagacagGTTTCAAACCCCGACCAGTT	KD screening
tdrd1_gRNA3	ttggagaccgcttctctgttgtgGTTTCAAACCCCGACCAGTT	KD screening
tdrd5_gRNA1	atcgtccgctcgttgctgatttcGTTTCAAACCCCGACCAGTT	KD screening
tdrd5_gRNA2	aggccatcggatttacattcatgGTTTCAAACCCCGACCAGTT	KD screening
tdrd5_gRNA3	atggaggactgtctgttcatgacGTTTCAAACCCCGACCAGTT	KD screening
tdrd6_gRNA1	cttgcatcctctttgtgtgcttgGTTTCAAACCCCGACCAGTT	KD screening
tdrd6_gRNA2	ctgtgtcctgaaatgattggctgGTTTCAAACCCCGACCAGTT	KD screening
tdrd6_gRNA3	ctctgagggagtttactctgtaaGTTTCAAACCCCGACCAGTT	KD screening
tdrd7a_gRNA1	gaggcatgctattttgcatcaggGTTTCAAACCCCGACCAGTT	KD screening
tdrd7a_gRNA2	atgtggaccacggctttttcgagGTTTCAAACCCCGACCAGTT	KD screening
tdrd7a_gRNA3	tcaggcgtctgtggaggtttttgGTTTCAAACCCCGACCAGTT	KD screening
tdrd7b_gRNA1	ggggttcctaggttttctctgaaGTTTCAAACCCCGACCAGTT	KD screening
tdrd7b_gRNA2	gagcggaccgagttatttatcctGTTTCAAACCCCGACCAGTT	KD screening
tdrd7b_gRNA3	aggtcctcttcatagactttgctGTTTCAAACCCCGACCAGTT	KD screening
tdrd9_gRNA1	cacaggcaaaagctaatttctttGTTTCAAACCCCGACCAGTT	KD screening
tdrd9_gRNA2	ctggacgaccttcataaactgttGTTTCAAACCCCGACCAGTT	KD screening
tdrd9_gRNA3	actgttcctgatgtcaaatatgtGTTTCAAACCCCGACCAGTT	KD screening
tdrd12_gRNA1	ctggggacttcaagatctgtgtcGTTTCAAACCCCGACCAGTT	KD screening
tdrd12_gRNA2	gggtgggtcttgatgaagaattaGTTTCAAACCCCGACCAGTT	KD screening
tdrd12_gRNA3	ctggcatggcttcttattatagtGTTTCAAACCCCGACCAGTT	KD screening
ddx4_gRNA1	atgggcatcaacttcgacaagtaGTTTCAAACCCCGACCAGTT	KD screening
ddx4_gRNA2	aaggtggactacctgtttgtggcGTTTCAAACCCCGACCAGTT	KD screening
ddx4_gRNA3	tccagcacgtggtgaactttgacGTTTCAAACCCCGACCAGTT	KD screening
ddx19_gRNA1	gttggaggctctctataatctctGTTTCAAACCCCGACCAGTT	KD screening
ddx19_gRNA2	ctcggcagttggactcttctgaaGTTTCAAACCCCGACCAGTT	KD screening
ddx19_gRNA3	ttgaagggggtctatacgatgggGTTTCAAACCCCGACCAGTT	KD screening
dazl_gRNA1	cgggttcgtgtacttcaatgaagGTTTCAAACCCCGACCAGTT	KD screening
dazl_gRNA2	gttggagggatcgacatgaaggtGTTTCAAACCCCGACCAGTT	KD screening
dazl_gRNA3	gctgggccctgctattttgaaagGTTTCAAACCCCGACCAGTT	KD screening
piwil1_gRNA1	tgatggagcgttgctcattttgcGTTTCAAACCCCGACCAGTT	KD screening
piwil1_gRNA2	cgcggacatttcattcatgaactGTTTCAAACCCCGACCAGTT	KD screening
piwil1_gRNA3	gtgctacctaacaggtttgactgGTTTCAAACCCCGACCAGTT	KD screening
piwil2_gRNA1	gaggatctgttctgtcaagtctaGTTTCAAACCCCGACCAGTT	KD screening
piwil2_gRNA2	ctcgtgtccatgtttagaggaatGTTTCAAACCCCGACCAGTT	KD screening
piwil2_gRNA3	tgatggctccattctgtatctgcGTTTCAAACCCCGACCAGTT	KD screening
mov10L_gRNA1	catggtgtccaaacttttgtctcGTTTCAAACCCCGACCAGTT	KD screening
mov10L_gRNA2	gtctgacggttatattccaatgaGTTTCAAACCCCGACCAGTT	KD screening
mov10L_gRNA3	aggggctctggtattctggatacGTTTCAAACCCCGACCAGTT	KD screening
buc_gRNA1	ctgcctggaggttttaatgttggGTTTCAAACCCCGACCAGTT	KD screening
buc_gRNA2	ggagtctcctactgtgacttttgGTTTCAAACCCCGACCAGTT	KD screening
buc_gRNA3	gttgtgggaaaagtttgaataaaGTTTCAAACCCCGACCAGTT	KD screening
bucL_gRNA1	ctcagctccatttgatggactacGTTTCAAACCCCGACCAGTT	KD screening
bucL_gRNA2	gtgtgacgccaagctttcatctgGTTTCAAACCCCGACCAGTT	KD screening
bucL_gRNA3	gctggctgtctgactttgagaacGTTTCAAACCCCGACCAGTT	KD screening

elavl2_gRNA1	accggctggtgcattttcgtctaGTTTCAAACCCCGACCAGTT	KD screening
elavl2_gRNA2	aggctatggatttgttaactatgGTTTCAAACCCCGACCAGTT	KD screening
elavl2_gRNA3	aggttggacaatctgttgaacatGTTTCAAACCCCGACCAGTT	KD screening
gdf3_gRNA1	gcggtaacatcattcgatacgttGTTTCAAACCCCGACCAGTT	KD screening
gdf3_gRNA2	acggcttggttttagaactgcgtGTTTCAAACCCCGACCAGTT	KD screening
gdf3_gRNA3	cagcgtggttactcgttgctggtGTTTCAAACCCCGACCAGTT	KD screening
gpat2_gRNA1	tgctgtgccctgtttgtgagtggGTTTCAAACCCCGACCAGTT	KD screening
gpat2_gRNA2	tctgctggtttatgtgtacttgcGTTTCAAACCCCGACCAGTT	KD screening
gpat2_gRNA3	gtgggcatctcttatgactctgtGTTTCAAACCCCGACCAGTT	KD screening
gasz_gRNA1	ggagtccacttatgtttgctgtcGTTTCAAACCCCGACCAGTT	KD screening
gasz_gRNA2	cagatgacctctttgatgcttgcGTTTCAAACCCCGACCAGTT	KD screening
gasz_gRNA3	tgcgtgtggtttacaatgggtgtGTTTCAAACCCCGACCAGTT	KD screening
golga3_gRNA1	atggtcctataacatctgctttgGTTTCAAACCCCGACCAGTT	KD screening
golga3_gRNA2	tttggctgattataaatctccagGTTTCAAACCCCGACCAGTT	KD screening
golga3_gRNA3	actggagtctttgtcatctgaggGTTTCAAACCCCGACCAGTT	KD screening
rbm46_gRNA1	gctggctctgatggagaagacggGTTTCAAACCCCGACCAGTT	KD screening
rbm46_gRNA2	cgtggtggacgtcatcgtgtatcGTTTCAAACCCCGACCAGTT	KD screening
rbm46_gRNA3	ctgctgctgctctacaaggtggtGTTTCAAACCCCGACCAGTT	KD screening
rnf17_gRNA1	tactgcgtcctggataagttgttGTTTCAAACCCCGACCAGTT	KD screening
rnf17_gRNA2	ctgctctggaaatggctgttcttGTTTCAAACCCCGACCAGTT	KD screening
rnf17_gRNA3	cttggaggctgtgaaaatctgtcGTTTCAAACCCCGACCAGTT	KD screening
rnf213a_gRNA1	ttggagctcctcaggtataaaatGTTTCAAACCCCGACCAGTT	KD screening
rnf213a_gRNA2	ttgggacacccttgttctgtgctGTTTCAAACCCCGACCAGTT	KD screening
rnf213a_gRNA3	tctgggcctgtctgttttcatggGTTTCAAACCCCGACCAGTT	KD screening
rnf213b_gRNA1	attgcggtcacattattgtggagGTTTCAAACCCCGACCAGTT	KD screening
rnf213b_gRNA2	gctgacgcatgatgaatcaacatGTTTCAAACCCCGACCAGTT	KD screening
rnf213b_gRNA3	tggagaccatatttgatctgcttGTTTCAAACCCCGACCAGTT	KD screening
smc1b_gRNA1	ttggcgtgggaagcatgttatcgGTTTCAAACCCCGACCAGTT	KD screening
smc1b_gRNA2	cctcgctgagctttactataatgGTTTCAAACCCCGACCAGTT	KD screening
smc1b_gRNA3	gtgggagctgaaagcaaacagtgGTTTCAAACCCCGACCAGTT	KD screening
taf4b-gRNA1	ctgtccttctgttatgctgagtgGTTTCAAACCCCGACCAGTT	KD screening
taf4b-gRNA2	tggtgactctgatcaagctggctGTTTCAAACCCCGACCAGTT	KD screening
taf4b-gRNA3	tgcggtgcgttcactgacatcagGTTTCAAACCCCGACCAGTT	KD screening
topaz_gRNA1	aatggctgctttgactctgtctgGTTTCAAACCCCGACCAGTT	KD screening
topaz_gRNA2	atgtggcgtcattttacctaaagGTTTCAAACCCCGACCAGTT	KD screening
topaz_gRNA3	cggctgtactgtctgaaggtttaGTTTCAAACCCCGACCAGTT	KD screening
adad1_gRNA1	ggagcctgttttgcacaaaatctGTTTCAAACCCCGACCAGTT	KD screening
adad1_gRNA2	tgcagaagctctgttttgcctttGTTTCAAACCCCGACCAGTT	KD screening
adad1_gRNA3	atgctgcaccgctttaaacttgtGTTTCAAACCCCGACCAGTT	KD screening

Table S5. Primers used for vector construction, in vitro transcription (IVT), knock-in (KI) donor DNA synthesis, qPCR, and XX-XY genotyping, related to Figures S1 and S3 and STAR Methods

primer name	5'-sequence-3'	Note
Cas9_mSA_IVT_F+T3	acgccaagctcgaaattaaccctcactaaagggagctacttgttctttttgcaggatcc	PCR amplification of Cas9-mSA template for IVT (T3 promoter)
Cas9_IVT_R	caaaagctgggtaccgggcc	PCR amplification of Cas9-mSA template for IVT (T3 promoter)
T3_SC_F	acgccaagctcgaaattaaccctcactaaagggcgaattgggtaccgtat	PCR amplification of germ plasm genes for IVT (T3 promoter)
SV40_R_KpnI	gccggtaccacgccttaagatacattgat	PCR amplification of germ plasm genes for IVT (T3 promoter)
nanos3_F_BamHI	catggatccatgtcagacgtggagtctgg	Construction of *nanos3* expressing vector by GoldenGATE cloninig
nanos3(+)_R_KpnI	gccggtacctcaacgcctagacttcacat	Construction of *nanos3* expressing vector by GoldenGATE cloninig
dnd1_F_BamHI	ccggatccatggataaccaaagcaaagt	Construction of *dnd1* expressing vector by GoldenGATE cloninig
dnd1(+)_R_KpnI	gccggtacctcaggcgctcaaaccgctgc	Construction of *dnd1* expressing vector by GoldenGATE cloninig
nanos3_mut_F	TtcTcgGttCcaCctAtggaaAgactacatgggcctgtcgga	Introduction of silent mutations in the gRNA target site of *nanos3*
nanos3_mut_R	TttccaTagGtgGaaCcgAgaActgcttggctccatgatcag	Introduction of silent mutations in the gRNA target site of *nanos3*
dnd1_mut_F	TgcGgtAgtAgcAttTtcAtctcatcatgcagcctccat	Introduction of silent mutations in the gRNA target site of *dnd1*
dnd1_mut_R	TgaAaaTgcTacTacCgcAgacaccccctctatgccagg	Introduction of silent mutations in the gRNA target site of *dnd1*
SV40_F_BamHI	catggatccctgatcataatcagccatacc	Construction of *nanos3*- and *dnd1-SV40pA* by GoldenGATE cloninig
SV40_R_KpnI	gccggtaccacgccttaagatacattgat	Construction of *nanos3*- and *dnd1-SV40pA* by GoldenGATE cloninig
nanos3_3'UTR_F_BamHI	gccggatccaaagactcaatccattgtaa	Construction of *nanos3-nanos3* 3' UTR by GoldenGATE cloninig
nanos3_3'UTR_R_KpnI	gccggtaccgcaaccaaagacaacctggt	Construction of *nanos3-nanos3* 3' UTR by GoldenGATE cloninig
dnd_3'UTR_F_BamHI	ccggatccacattttagtgcctgtgtgt	Construction of *dnd1-dnd1* 3' UTR by GoldenGATE cloninig
dnd_3'UTR_R_KpnI	gccggtaccgaaagcagagatttaatttttatgggtctggtg	Construction of *dnd1-dnd1* 3' UTR by GoldenGATE cloninig
vasa_F+15	accgtatccggatccatggacgactgggaggaagaggaa	Construction of *vasa-SV40pA* by NEBuilder® HiFi DNA Assembly
vasa_R+15	gctgattatgatcagtcactcccagtcatcttcatcagcag	Construction of *vasa-SV40pA* by NEBuilder® HiFi DNA Assembly
piwil1_F+15	accgtatccggatccatgtctggtcgggctcgtgc	Construction of *piwil1-SV40pA* by NEBuilder® HiFi DNA Assembly
piwil1_R+15	gctgattatgatcagtcacaggtagaagaggtagt	Construction of *piwil1-SV40pA* by NEBuilder® HiFi DNA Assembly
tdrd6_F+15	accgtatccggatccatgtcttccatccagggtatgc	Construction of *tdrd6-SV40pA* by NEBuilder® HiFi DNA Assembly
tdrd6_R+15	gctgattatgatcagctacttctctgtaagcttctcag	Construction of *drd6-SV40pA* by NEBuilder® HiFi DNA Assembly
tdrd7a_F+15	accgtatccggatccatggagaacgagtcaattga	Construction of *tdrd7a-SV40pA* by NEBuilder® HiFi DNA Assembly
tdrd7a_R+15	gctgattatgatcagtcactcgatgtctgccatga	Construction of *tdrd7a-SV40pA* by NEBuilder® HiFi DNA Assembly
dazl_F+15	accgtatccggatccatggatctgaacagacccag	Construction of *dazl-SV40pA* by NEBuilder® HiFi DNA Assembly
dazl_R+15	gctgattatgatcagctacagcaccgttcgcatag	Construction of *dazl-SV40pA* by NEBuilder® HiFi DNA Assembly
buc_F+15	accgtatccggatccatggatgattcaagcaagcagc	Construction of *buc-SV40pA* by NEBuilder® HiFi DNA Assembly
buc_R+15	gctgattatgatcagtcaacatctgggatctgatctgc	Construction of *buc-SV40pA* by NEBuilder® HiFi DNA Assembly
dazap2_F+15	accgtatccggatccatgaataacaaaggttcatatccacagcaggc	Construction of *dazap2-SV40pA* by NEBuilder® HiFi DNA Assembly
dazap2_R+15	gctgattatgatcagttaccagatggtataccctccat	Construction of *dazap2-SV40pA* by NEBuilder® HiFi DNA Assembly
SV40pA_F_vec_amp	ctgatcataatcagccataccacatttgtagag	Linearization of pGGDestSC-SV40pA by PCR
SC(-)_R_vec_amp	ggatccggatacggtaccca	Linearization of pGGDestSC-SV40pA by PCR
Znanos3_F_BamHI	catggatccatggctttttctcttctcca	Construction of zebrafish *nanos3-SV40pA* by GoldenGATE cloninig
Znanos3_R_KpnI	catggtacctcaccatgttgatttggcgt	Construction of zebrafish *nanos3-SV40pA* by GoldenGATE cloninig
Zdnd_F_BamHI	catggatccatggtcggagacatggatgc	Construction of zebrafish *dnd1-SV40pA* by GoldenGATE cloninig
Zdnd_R_KpnI	catggtaccttagaaaggccgtaaatttg	Construction of zebrafish *dnd1-SV40pA* by GoldenGATE cloninig
M13f_vasa_5HF_FL_F	GTAAAACGACGGCCAGTtgcagcgccccctgctgctgctgatgaagatgactgggagCTGAGCGGTGGAGGCGGTTC	1st PCR amplification for 5' biotinylated donor DNA synthesis
M13r_vasa_3HF_EGFP_R	CAGGAAACAGCTATGACgcattcagtggaggaggaagctgacgcggcggggccttcaCTTGTACAGCTCGTCCATGC	1st PCR amplification for 5' biotinylated donor DNA synthesis
5' bion_M13_F	/5BiosG/GTAAAACGACGGCCAGT	2nd PCR amplification for 5' biotinylated donor DNA synthesis
5' bion_M13_R	/5BiosG/CAGGAAACAGCTATGAC	2nd PCR amplification for 5' biotinylated donor DNA synthesis
vasa_gRNA_F	taatacgactcactatagggaagatgactgggagtga	PCR amplification of *vasa*-gRNA template for IVT (T7 promoter)
vasa_gRNA_R	ttctagctctaaaactcactcccagtcatcttc	PCR amplification of *vasa*-gRNA template for IVT (T7 promoter)
vasa:EGFP_KI_seq_F1	gcctccacagactccaggaag	Sequence analysis of *vasa:EGFP* KI allele (seq1)
vasa:EGFP_KI_seq_R1	cctcgcccttgctcaccat	Sequence analysis of *vasa:EGFP* KI allele (seq1)
vasa:EGFP_KI_seq_F2	gcccgacaaccactacctga	Sequence analysis of *vasa:EGFP* KI allele (seq2)
vasa:EGFP_KI_seq_R2	tttgttgaaaacttttatttatcaggagaaaaccgct	Sequence analysis of *vasa:EGFP* KI allele (seq2)
piwil1_probe_F	ggccaggctactctaccacc	DIG probe for in situ hybridization
piwil1_probe_R_T7	GGCCAGTGAATTGTAATACGACTCACTATAGGGagcagtcaatccccacaatc	DIG probe for in situ hybridization
tdrd1_probe_F	tcagaacctggtggacggtg	DIG probe for in situ hybridization
tdrd1_probe_R_T7	GGCCAGTGAATTGTAATACGACTCACTATAGGGgggagagcacgttagcagag	DIG probe for in situ hybridization
cxcr4b_probe_F	atggagtatttctatgaaagcatcgtt	DIG probe for in situ hybridization
cxcr4b_probe_R_T7	GGCCAGTGAATTGTAATACGACTCACTATAGGGttaacttgacaaaacactcgaagact	DIG probe for in situ hybridization
dazl_probe_F	acgcgggggtgataggcgtg	DIG probe for in situ hybridization
dazl_probe_R_T7	GGCCAGTGAATTGTAATACGACTCACTATAGGGctacagcaccgttcgcatag	DIG probe for in situ hybridization
q-nanos3_mut_F	TtcTcgGttCcaCctAtggaaAg	q-RT-PCR
q-nanos3_R	ccgcactccaggtaggactc	q-RT-PCR
q-dnd1_mut_F	gtgtcTgcGgtAgtAgcAttTtcA	q-RT-PCR
q-dnd1_R	tctctgcagagagccacttgatag	q-RT-PCR
q-EGFP_F	gcccgacaaccactacctga	q-RT-PCR
q-EGFP_R	gtccatgccgagagtgatcc	q-RT-PCR
dmy-FAM	TGGCTTCACCGTTGGA	TaqMan MGB probe for XX-XY genotyping
cyp19a-VIC	ACAACAAATATGGAGACATT	TaqMan MGB probe for XX-XY genotyping
tq-dmy-F	CGGTAAATTGACGCACAGCAT	XX-XY genotyping
tq-dmy-R	TCCAGTAAGTTGCAGAGTTTCGTT	XX-XY genotyping
tq-cyp19a-F	TGGCACAGCCAGCAACTATTA	XX-XY genotyping
tq-cyp19a-R	TCCGTTGATCCACACTCGAA	XX-XY genotyping
